# Oncogenes and tumor suppressor genes: functions and roles in cancers

**DOI:** 10.1002/mco2.582

**Published:** 2024-05-31

**Authors:** Tikam Chand Dakal, Bhanupriya Dhabhai, Anuja Pant, Kareena Moar, Kanika Chaudhary, Vikas Yadav, Vipin Ranga, Narendra Kumar Sharma, Abhishek Kumar, Pawan Kumar Maurya, Jarek Maciaczyk, Ingo G. H. Schmidt‐Wolf, Amit Sharma

**Affiliations:** ^1^ Department of Biotechnology Genome and Computational Biology Lab Mohanlal Sukhadia University Udaipur Rajasthan India; ^2^ Department of Biochemistry Central University of Haryana Mahendergarh Haryana India; ^3^ School of Life Sciences. Jawaharlal Nehru University New Delhi India; ^4^ Dearptment of Agricultural Biotechnology DBT‐NECAB, Assam Agricultural University Jorhat Assam India; ^5^ Department of Bioscience and Biotechnology Banasthali Vidyapith Tonk Rajasthan India; ^6^ Manipal Academy of Higher Education Manipal Karnataka India; ^7^ Institute of Bioinformatics, International Technology Park Bangalore India; ^8^ Department of Stereotactic and Functional Neurosurgery University Hospital of Bonn Bonn Germany; ^9^ Department of Integrated Oncology Center for Integrated Oncology (CIO) University Hospital Bonn Bonn Germany

**Keywords:** cancer signaling, cancer therapy, ncRNAs, tumor suppressor genes (TSGs), X chromosome–autosome crosstalk, X‐chromosome inactivation

## Abstract

Cancer, being the most formidable ailment, has had a profound impact on the human health. The disease is primarily associated with genetic mutations that impact oncogenes and tumor suppressor genes (TSGs). Recently, growing evidence have shown that X‐linked TSGs have specific role in cancer progression and metastasis as well. Interestingly, our genome harbors around substantial portion of genes that function as tumor suppressors, and the X chromosome alone harbors a considerable number of TSGs. The scenario becomes even more compelling as X‐linked TSGs are adaptive to key epigenetic processes such as X chromosome inactivation. Therefore, delineating the new paradigm related to X‐linked TSGs, for instance, their crosstalk with autosome and involvement in cancer initiation, progression, and metastasis becomes utmost importance. Considering this, herein, we present a comprehensive discussion of X‐linked TSG dysregulation in various cancers as a consequence of genetic variations and epigenetic alterations. In addition, the dynamic role of X‐linked TSGs in sex chromosome–autosome crosstalk in cancer genome remodeling is being explored thoroughly. Besides, the functional roles of ncRNAs, role of X‐linked TSG in immunomodulation and in gender‐based cancer disparities has also been highlighted. Overall, the focal idea of the present article is to recapitulate the findings on X‐linked TSG regulation in the cancer landscape and to redefine their role toward improving cancer treatment strategies.

## Graphical abstract

X chromosome showing TSGs position (right) and functional effects of tumorigenic miRNAs (onco‐miRs) and tumor‐inhibitory miRNAs (left).

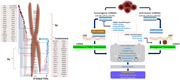

## 1 INTRODUCTION

All cancers are rooted to mutations in oncogenes (OCGs). OCGs can be defined as a modified version of a proto‐OCG, a class of genes involved in normal cell division and growth but carry some deleterious mutations. An OCG is formed when a proto‐OCG is altered extensively to produce excessive amounts of its copies or increase its level of activity over normal. Consequently, growth control is lost due to defects in different regulatory systems, which alter cell behavior and uncontrolled multiplication of cancer cells that eventually invade normal tissues and organs and spread cancer throughout the body. Cancer development has also been thought to involve the selection for cells with greater proliferation, survival, invasion, and metastasis. On the contrary, tumor suppressor genes (TSGs) are genes that regulate cell division and apoptosis under normal conditions. Dysregulated TSGs can result in uncontrolled cell growth, potentially causing cancer. While OCGs, when altered, result in gain‐of‐function activity, TSGs lead to loss‐of‐function activity, both of which contribute to the development of cancer. Both OCGs and deregulated TSGs cause aberrant signal transmission in cancer leading to uncontrolled cell multiplication, metastasis, apoptotic loss, and angiogenesis. Functional behaviors of both OCGs and TSGs are intricate and require additional research to completely clarify cancer pathways and carcinogenesis. This review examines the prevalent anomalies in OCGs and TSGs in different cancer and their correlations with clinical outcomes such as tumor categorization, prognosis, and response to particular treatments.

Neoplasms result from acquired and physical genetic changes in proto‐OCGs, tumor‐suppressor genes, and DNA‐repair genes.^1^ One contributing factor is the genetic induction resulting from the modification of gene regulators,^2^ inactivation of TSGs,^3^, ^4^ genetic mutations,^5^, ^6^ protein modifications,^7^, ^8^ and epigenetic alterations.^9^, ^10^ These factors collectively lead to the aberrant proliferation and growth of cells. The proper regulation of these genes is capable of governing accurate transcriptional activity, gene expression, and gene silencing. The interplay between the genome and epigenome is a significant factor in the progression of cancer. Enhanced comprehension of these interplays would result in novel therapeutics as well.

The identification of dominant “activating” OCGs has led to the assumption that a unique class of “suppressor genes” may prevent cancers. Specifically, the alteration of a small number of genes with oncogenic and tumor‐suppressor properties is principally responsible for the restructuring of the cancer genome. Somatic cell fusion and chromosome separation research have found tumorigenicity‐inhibiting genes.^11^ Carcinogenesis is complicated and caused by OCG function or TSG mutations.^12^ Most of our knowledge of TSGs comes from the preliminary investigation of retinoblastoma (RB) genes, the first TSG discovered, and the mutation that causes it in children.^13^, ^14^ RB susceptibility gene (Rb1) gene inactivation causes this hereditary illness. Rb1 gene inactivation mutation raises the incidence of eye RB 10,000‐fold more than the general population.^15^ As such, cancer represents a significant medical challenge within contemporary civilizations, clinicians, and medical practitioners. In light of this, TSGs are obviously of special interest in relation to malignancies, as their inactivation can lead to uncontrolled cell division.

Cancers start with one cell, according to the clonal theory while the malignant tumors are clonally generated; however, it does not mean they are caused by one mutation. OCGs often work together to cause cancer. Genetically engineered mice with the *RAS* and *MYC* OCGs show the combined effect of two cancer‐causing genes. Only a percentage of transgenic mice developed cancer from the *MYC* OCG after 100 days. The *MYC* OCG was first found in the avian retrovirus genome and activated in cancer cells via proviral integration, gene amplification, and chromosomal translocation. The *RAS* OCG caused tumorigenesis earlier than the *MYC* OCG. After 150 days, 50% of transgenic mice developed tumors.^16^ Cancer developed faster in all transgenic mice with RAS and MYC OCGs. Cancer cells employ the Knudson's two‐hit process to effectively silence autosomal TSGs. This technique involves the sequential occurrence of loss‐of‐function mutations followed by loss of heterozygosity (LOH) at the specific loci of the TSGs. Nevertheless, the discovery of X‐linked TSGs has posed a challenge to the conventional “two‐hit inactivation” paradigm in TSGs. This has introduced a new perspective, suggesting that a solitary genetic alteration can result into the loss of suppressor activity. TSGs contain a number of insertions, deletions, missense/nonsense mutations, frame‐shift mutations as well as epigenetic changes that render a protein inactive. Most TSGs, including *RB*, require the inactivation of both alleles for carcinogenesis.^17^ A single mutation at one allele may be sufficient in other TSGs, for instance, *TP53* and *PTEN*, to result in an altered cell phenotype and a reduced degree of tumor suppressor activity during the initiation and progression of tumors.^18^, ^19^ The X‐linked genes only have one allele in males, and one allele is rendered inactive in each female cell as a means of dosage compensation. Since X chromosome inactivation (XCI) serves as a functional LOH for X‐linked TSGs,^20^, ^21^ these genes are therefore more vulnerable to genetic damages that encourage tumor formation and progression.^22^, ^23^, ^24^ Additionally, a single genetic hit is sufficient to inactivate an X‐linked TSG.^24^

The precise mechanism by which these genes are suppressed in human cancer remains uncertain; nonetheless, gaining a comprehensive grasp of these intricacies will significantly contribute to our comprehension of the development and progression of human cancer. Recent data have demonstrated that X‐linked TSGs have a distinct function in the progression and spread of cancer. Approximately 6% of our genome consists of genes that serve as tumor suppressors. Notably, the X chromosome contains a significant amount of these TSGs. The situation gets even more intriguing due to the presence of X‐linked TSGs on the X chromosome, which undergoes XCI. Hence, it is crucial to thoroughly investigate the novel framework concerning X‐linked TSGs, such as their interaction with autosomes, control by ncRNAs, genomic changes, mutational patterns, and other relevant factors. This article provides a comprehensive analysis of the dysregulation of TSGs on the X chromosome in different types of malignancies. The major and studied significance of X‐linked TSGs in the interaction between sex chromosomes and autosomes in the remodeling of the cancer genome is emphasized. The significance of noncoding areas in the control of tumor TSGs on the X chromosome as well as the involvement of TSGs in gender‐based disparities in cancer are emphasized. This review aims to generate novel hypotheses and insights for the identification and management of cancer, with a focus on precision medicine and personalized medicines.

In current review, we aim to comprehensively discuss what are OCGs, OCG‐driven cancers, TSGs, and X‐linked TSGs. We also discussed involvement of TSGs in cancers, process of TSGs inactivation, and X chromosome—autosome crosstalks. Following this, importance of X‐linked TSG mutants, variants, and polymorphisms in cancer initiation, progression, and metastasis has been reviewed. X‐Linked TSGs mediate immune response via a number of mechanisms. We also shed some lights on how X‐linked TSGs contribute to the gender‐based discrepancies in different cancers. Finally, implications of TSGs in cancer diagnosis, prognosis and clinical relevance have been highlighted. In the discussion and the conclusion section of the review, we concisely and comprehensively summarized TSGs related cell signaling pathways and their regulation and prospective advancements in TSG‐based cancer therapies.

## 2 AND OCG‐DRIVEN CANCERS

Proto‐OCGs are genes that often facilitate cellular growth and division with the purpose of generating new cells or promoting cell survival.^25^, ^26^ When a proto‐OCG undergoes a mutation or experiences gene amplification, it can become aberrantly activated, leading to its transformation into an OCG. OCGs, when activated, can promote the formation of cancer. Under such circumstances, the cell may undergo uncontrolled proliferation, potentially resulting in the development of cancer.^26^ OCGs can be triggered in cells through many mechanisms, for instance genetic variations/mutations, gene duplication, epigenetic changes, chromosomal rearrangements, and others.

Activation must be demonstrated in human cancer cases and experimental activation of the gene in a cell culture or animal model must be able to reproduce the malignancy to meet the rigorous criterion. Activation can be achieved by gene amplification, resulting in an increased amount of the protein produced by the gene, so enhancing its function. One instance of this method of OCG activation is HER‐2, present in approximately 20% of first breast cancer instances. Another way of activating is through a point mutation that boosts the function of the “oncoprotein.” Point mutations in the ras OCG are frequently observed in lung, colorectal, and pancreatic cancers, but not in breast cancer. Point mutations in ras codons 12, 13, and 61 hinder the interaction between p21ras and GTPase‐activating protein (GAP), resulting in the sustained activation of p21ras in a GTP‐bound state.^27^ This enhances downstream signaling processes such cell cycle activation. Another way OCGs can be activated is through chromosomal translocation, when a novel fusion gene is produced and translated into a protein that has increased activity. OCGs can also work together with other genetic or epigenetic alterations. There has been significant interest in the oncogenic aspects of cell signaling systems in breast cancer, such as the HER‐2/Neu cascade.^28^ The HER‐2 membrane receptor tyrosine kinase is a well‐researched element of this system, but various other proteins like Ras play a role in transmitting and adjusting this signal. The outcomes of this signal include cell proliferation, changes in drug response and DNA repair, angiogenesis, apoptosis, protease activity, and cell movement.

### 2.1 Characteristics and mechanism of activation of OCGs

Many OCGs have been identified in human malignancies; however, only a few number are considered essential for the advancement of cancer. Some OCGs can induce cancer in transgenic mouse models when they are overexpressed, and different OCGs result in unique characteristics in mice.^29^ Amplification and overexpression of OCGs and their products are the primary processes by which these genes contribute to cancer development. Amplification can range from small chromosomal segments to entire chromosomal arms, encompassing hundreds of genes, or even entire chromosomes. The descriptions provided are of OCGs and proto‐OCGs that are widely agreed upon to play a role in the development of cancer.

The activation mechanisms of proto‐OCGs could happen in four different ways. (1) Chromosomal translocation involves moving a proto‐OCG from a nontranscribable site to a nearby transcribable location, such as the *MYC* OCG in human Burkitt's lymphoma. (2) A point mutation in a proto‐OCG involves the substitution of a single base with another base, resulting in the replacement of an amino acid in the oncoprotein. For example, a point mutation occurring at codon 12 of the *RAS* OCG. (3) Gene amplification involves the insertion of several copies of an OCG, leading to elevated levels of oncoprotein synthesis, such as *c‐MYC* in neuroblastoma. (4) Introducing a promoter gene near a proto‐OCG can lead to the overexpression of the gene, as seen in the retrovirus carcinogenicity process.

### 2.2 OCG‐driven cancer development

Proto‐OCGs are essential regulatory factors in normal cells for biological activities. Proto‐OCGs can act as growth factors, cellular signal transducers, and nuclear transcription factors (TFs). Mammalian and avian genomes harbor many proto‐OCGs that regulate typical cell differentiation and proliferation. Alterations in these genes that affect their regulation or the structure of their encoded proteins can manifest as activated OCGs in cancer cells. OCGs, once created, stimulate cell proliferation and play a crucial part in the development of cancer. Physical mutations that activate proto‐OCGs can be categorized into two types: those that alter the structure of the encoded protein and those that disrupt protein expression regulation.^30^ Structural mutations involve point mutations in RAS proto‐OCGs and chromosomal translocations that create hybrid genes, including the Philadelphia translocation. Enhanced gene expression in human cancers can result from gene amplification or chromosome translocation, such as when the MYC gene is placed under the regulation of immunoglobulin enhancer sequences.^31^

Activation of proto‐OCGs leads to their transformation into OCGs; currently, 50–60 OCGs have been identified. While multiple proto‐OCGs have been found in an activated, oncogenic state in human tumor genomes, the specific genetic alterations responsible for these activations are yet unknown. Proto‐OCG expression in a typical cell is regulated by its own transcriptional promoter, which is a specific DNA sequence responsible for controlling transcription levels. Every proto‐OCG promoter allows the gene to respond to various physiological cues. A proto‐OCG can be expressed at modest levels based on the cell's metabolic requirements. However, under certain circumstances, the gene's expression can be significantly increased.

The majority of human malignancies exhibit deregulation of C‐MYC. Increased C‐MYC expression can result from either a disruption in the signal transduction system responsible for its expression or mutations in the C‐MYC locus. C‐MYC is the most frequently mutated gene in malignancies. Novel therapeutic strategies for cancer treatment focus on targeting C‐MYC due to its overexpression being a common cause of tumor formation. The C‐MYC OCG regulates a second mutation that disables the apoptotic pathway, such as p53, in cases where normal cell apoptotic pathways do not have sufficient survival factors. Apoptotic inhibition and cell survival are promoted by two synergistic OCGs, including BCL2, BCL‐xL, and RAS.^32^, ^33^

### 2.3 Targeted therapies for OCG‐driven cancers

The majority of targeted therapies aid in the treatment of cancer by inhibiting particular proteins that promote tumor growth and metastasis. This is in contrast to chemotherapy, which frequently eradicates rapidly dividing and proliferating cells. The subsequent section describes the various methods by which targeted therapy treats cancer. Typically, targeted therapies consist of small‐molecule medicines or monoclonal antibodies. Small‐molecule medicines can quickly penetrate cells and are therefore suitable for targeting intracellular sites. Monoclonal antibodies, or therapeutic antibodies, are proteins created in a laboratory setting. The proteins are engineered to bind to particular targets present on cancer cells. Monoclonal antibodies target cancer cells to enhance their visibility and elimination by the immune system. Some monoclonal antibodies inhibit the growth of cancer cells or induce their self‐destruction. Some individuals transport poisons to cancerous cells.

There are a number of different targeted approaches available for treatment of tumors. (1) approaches that assist the immune system in killing cancerous cells, (2) approaches that impede the proliferation of cancer cells by disrupting the signals that initiate their haphazard division and growth, (3) cease signals that aid in blood vessel formation (angiogenesis), (4) transmit lethal substances to malignant cells for their effective killing, (5) induce apoptosis in cancer cells, and (6) deny cancer the hormones, growth factors and other cytokines/chemokines necessary for their proliferation.

Recent therapeutic strategies for the treatment of cancer are focusing on C‐MYC, as its overexpression is frequently implicated in the development of the disease. C‐MYC OCG regulates a second mutation that deactivates the apoptotic pathway (e.g., p53) in cells where adequate survival factors are absent from the apoptotic pathways of healthy cells. Cell survival and apoptosis inhibition are promoted by two synergistic OCGs, including BCL2, BCL‐xL, and RAS.^32^, ^33^

In the development of novel pharmaceuticals, such as antibodies and small synthetic molecules, targeting OCGs and their associated pathways has proven to be a promising strategy, according to recent therapeutic applications.^34^ Consequently, a more comprehensive comprehension of OCGs and TSGs through the lens of networks will yield innovative perspectives on their roles in the development of tumors. To our knowledge, however, no report investigates their relationships in a systematic fashion.

Antibodies to growth factor receptors were shown to inhibit growth in several preclinical models.^35^ Trastuzumab is a humanized recombinant monoclonal antibody directed against the extracellular portion of the HER‐2 protein.^36^ The mechanism of action of trastuzumab from animal models is presumed to be modulatory effects on cell signaling, but there is also evidence of an immunological effect.^37^ Response rates of 11−26% were seen when trastuzumab was used as a single agent, and this activity was higher (35%) in patients who, in retrospect, had truly HER‐2+ tumors by updated immunohistochemical or gene amplification criteria.^38^, ^39^, ^40^, ^41^ Greater activity was observed when trastuzumab was combined with chemotherapy, with the pivotal randomized trial showing improvements in response rate, time to disease progression, duration of response, and survival.^38^, ^39^, ^40^, ^41^

## 3 TSGs, X‐LINKED TSGs, AND THEIR INVOLVEMENT IN CANCERS

Tumor suppressors are genes that typically regulate process of normal cell growth and apoptosis. In particular, these genes inhibit growth and other processes that can impact invasive and metastatic capabilities, like cell adhesion and protease activity modulation. TSGs when not functioning properly, lead to the development of cancer. While hereditary anomalies are responsible for a small portion of breast cancer instances, these germline mutations are found in TSGs. The same genes can include sporadic acquired somatic mutations. Both scenarios involve a mutation in one allele and a deletion of the other allele, following Alfred Knudson's “two‐hit” hypothesis regarding RB.^42^ This hypothesis suggests that both gene alleles must be lost for the malignant phenotype to be revealed. There are instances where a mutation of the TSG may not occur, but instead, another mechanism disrupts its expression or function. This could involve gene promoter methylation inhibiting transcription, accelerated proteasomal breakdown, or irregularities in other proteins interacting with the gene product.

### 3.1 Characteristics and mechanism of inactivation of TSGs

Tumor‐suppressor genes share a similar characteristic: each gene has a role in protecting the organism from cancer. A cancer cell requires both copies of a tumor‐suppressor gene to be inactive in order to proliferate or survive. Mutations in tumor‐suppressor genes can result in cancer. Tumor‐suppressor genes are dispersed across the human genome and are involved in the development of different forms of human neoplasia. LOH can result from partial deficiencies, chromosome deletions, or irregular cell divisions, indicating the absence of one or more tumor‐suppressor genes.^43^ Microsatellite DNA is distinct due to its linear replication of smaller subunits (up to six nucleotides) and its lack of protein‐coding function. Microsatellite DNA is utilized in genetic research. Microsatellite DNA instability is linked to a higher mutation rate in microsatellite DNA sequences and the overall genome, which is connected to DNA‐repair system damage.^43^

TSGs have limited utility in diagnostic applications, except for inherited susceptibility genes. Efforts to replace the lost gene function through therapeutic techniques have been hindered by the technological challenges of effective gene delivery. TSGs encode proteins that often work to inhibit cellular growth and division or even facilitate programmed cell death (apoptosis), in contrast to the cell cycle‐promoting role of proto‐OCGs and OCGs.^25^, ^26^ Examples encompass proteins that impede the course of cell division, elements implicated in the preservation of cell division control points, and proteins necessary for the initiation of programmed cell death. One extensively researched element in this category is a protein called RB protein (pRb) and its related gene, *RB1*, which was the initial TSG to be discovered.^44^ When pRb activity is halted, the genes necessary for advancing into the S phase of the cell cycle are no longer expressed. Consequently, the deactivation of pRb leads to unregulated cell division. Indeed, this approach is applicable to all tumor suppressors: genetic modifications in the gene result in carcinogenesis, which hinders the regulatory protein's ability to restrain cell proliferation.^45^, ^46^ The most common genetic changes that result in the inactivation of pRb are frameshifts or deletions in the *RB1* gene, which lead to the early introduction of a stop codon and the production of a faulty protein. Occasionally, the expression of pRb may be intact, but the functionality of the route it operates in is impaired due to the inactivity of other components of the pathway.

The p53 gene is a prominent example of a tumor suppressor that is frequently altered in human cancers.^47^ P53 is a TF that triggers the production of proteins that restrict cell growth and promote cell death when DNA damage occurs. It is crucial for maintaining the G1 to S cell cycle checkpoint. Disabling p53 genetic mutations will impede the DNA damage response, which is responsible for halting cell cycle progression. During this process, a cell undergoes continued division despite the presence of DNA damage. Tumor suppressors are genes that, when inactivated, lose their ability to function properly. In order for tumorigenesis to happen, both the maternal and paternal copies of a gene coding for a tumor suppressor usually need to be altered. However, if one copy of the gene remains intact, it can still provide enough activity for the cell to maintain normal growth and division. While dominant gain‐of‐function mutations activate proto‐OCGs, while recessive loss‐of‐function mutations or epigenetic silencing inactivate TSGs. The human genome contains roughly 6% of TSGs and the X chromosome alone holds 2% (Table 1), we previously focused on X‐linked TSGs that appear to be implicated in 32 cancer types.^3^ Our analysis showed that (a) most X‐linked TSGs are involved in breast cancer dysregulation, followed by prostate cancer, and (b) despite escaping XCI, they still have altered promoter methylation linked to mutational burden, and (c) X‐linked TSGs (primarily q‐arm) interact spatially and genetically with autosomal loci.^3^ We proposed that X‐linked TSGs alone can significantly impact the dynamics of sex chromosome–autosome crosstalk to restructure the cancer genome, supporting our previous findings that loss/gain of entire sex chromosomes (in XO and XXY syndromes) can profoundly affect autosome epigenetic status.^3^

**TABLE 1:** Catalogue of 23 coding X‐linked TSGs in humans (sourced from: https://bioinfo.uth.edu/TSGene/?csrt=13635304675887717882).

S. No.	X‐linked TSGs	Ensembl ID	Gene description
1	BTK	ENSG00000010671	Bruton tyrosine kinase [Source:HGNC Symbol;Acc:HGNC:1133]
2	DDX3X	ENSG00000215301	DEAD‐box helicase 3 X‐linked [Source:HGNC Symbol;Acc:HGNC:2745]
3	DMD	ENSG00000198947	Dystrophin [Source:HGNC Symbol;Acc:HGNC:2928]
4	DUSP9	ENSG00000130829	Dual specificity phosphatase 9 [Source:HGNC Symbol;Acc:HGNC:3076]
5	FHL1	ENSG00000022267	Four and a half LIM domains 1 [Source:HGNC Symbol;Acc:HGNC:3702]
6	FLNA	ENSG00000196924	Filamin A [Source:HGNC Symbol;Acc:HGNC:3754]
7	GPC3	ENSG00000147257	Glypican 3 [Source:HGNC Symbol;Acc:HGNC:4451]
8	FOXO4	ENSG00000184481	Forkhead box O4 [Source:HGNC Symbol;Acc:HGNC:7139]
9	RBBP7	ENSG00000102054	RB binding protein 7, chromatin remodeling factor [Source:HGNC Symbol;Acc:HGNC:9890]
10	RPL10	ENSG00000147403	Ribosomal protein L10 [Source:HGNC Symbol;Acc:HGNC:10298]
11	KDM6A	ENSG00000147050	Lysine demethylase 6A [Source:HGNC Symbol;Acc:HGNC:12637]
12	ZNF185	ENSG00000147394	Zinc finger protein 185 with LIM domain [Source:HGNC Symbol;Acc:HGNC:12976]
13	SRPX	ENSG00000101955	Sushi repeat containing protein X‐linked [Source:HGNC Symbol;Acc:HGNC:11309]
14	TREX2	ENSG00000183479	Three prime repair exonuclease 2 [Source:HGNC Symbol;Acc:HGNC:12270]
15	RBMX	ENSG00000147274	RNA binding motif protein X‐linked [Source:HGNC Symbol;Acc:HGNC:9910]
16	RPS6KA6	ENSG00000072133	Ribosomal protein S6 kinase A6 [Source:HGNC Symbol;Acc:HGNC:10435]
17	FOXP3	ENSG00000049768	Forkhead box P3 [Source:HGNC Symbol;Acc:HGNC:6106]
18	TCEAL7	ENSG00000182916	Transcription elongation factor A like 7 [Source:HGNC Symbol;Acc:HGNC:28336]
19	EDA2R	ENSG00000131080	Ectodysplasin A2 receptor [Source:HGNC Symbol;Acc:HGNC:17756]
20	BCORL1	ENSG00000085185	BCL6 corepressor like 1 [Source:HGNC Symbol;Acc:HGNC:25657]
21	PHF6	ENSG00000156531	PHD finger protein 6 [Source:HGNC Symbol;Acc:HGNC:18145]
22	BEX2	ENSG00000133134	Brain expressed X‐linked 2 [Source:HGNC Symbol;Acc:HGNC:30933]
23	AMER1	ENSG00000184675	APC membrane recruitment protein 1 [Source:HGNC Symbol;Acc:HGNC:26837]

Several significant tumor suppressors in breast cancer have been identified, and more are being discovered. A hypothetical BRCA‐3 gene has been suggested for families with a significant history of breast cancer but no identified mutations in *BRCA‐1* or *BRCA‐2*. However, the exact location of this gene has not been determined.^48^

*PTEN* codes for a phosphatase that acts as an inhibitor of Akt. Loss of *PTEN* function enhances the Akt cell survival signal.^49^ PTEN mutations inherited in Cowden syndrome have been demonstrated to elevate the likelihood of breast and ovarian malignancies; however, sporadic incidences of gene mutation are infrequent.^50^, ^51^

*CHK2* is a serine threonine kinase that is mutated in certain families with a high risk of breast cancer and exhibits a Li‐Fraumeni syndrome phenotype, while having normal *TP53*, *BRCA‐1*, and *BRCA‐2* sequences.^52^ The kinase is activated by the ATM protein in response to DNA damage and subsequently phosphorylates p53 and BRCA‐1. A single truncating mutation was identified in 1% of a group of Finnish patients. Due to the frequency of breast cancer in this group, it is classified as a gene with low susceptibility to breast cancer.^53^

The *ATM* gene detects DNA damage and triggers checkpoints and DNA repair pathways by quickly phosphorylating several substrates such as p53, BRCA‐1, and CHK2.^54^ When both copies of the *ATM* gene are lost, it leads to ataxia‐telangiectasia, a condition characterized by gradual degeneration of the cerebellum, fragile blood vessels, immune system deficiencies, and increased risk of lymphoid cancers. There is a disagreement concerning whether individuals in the carrier state, which comprises roughly 1−2% of the population, are at a higher risk for breast cancer and DNA damage due to radiation. Estimates of cancer risk in individuals with one copy of a mutated gene vary, with some variants showing a risk up to 12 times greater, indicating that the risk might be influenced by the specific type of mutation.^55^

### 3.2 X‐linked TSGs‐ dosage compensation and LOH

**FIGURE 1:**
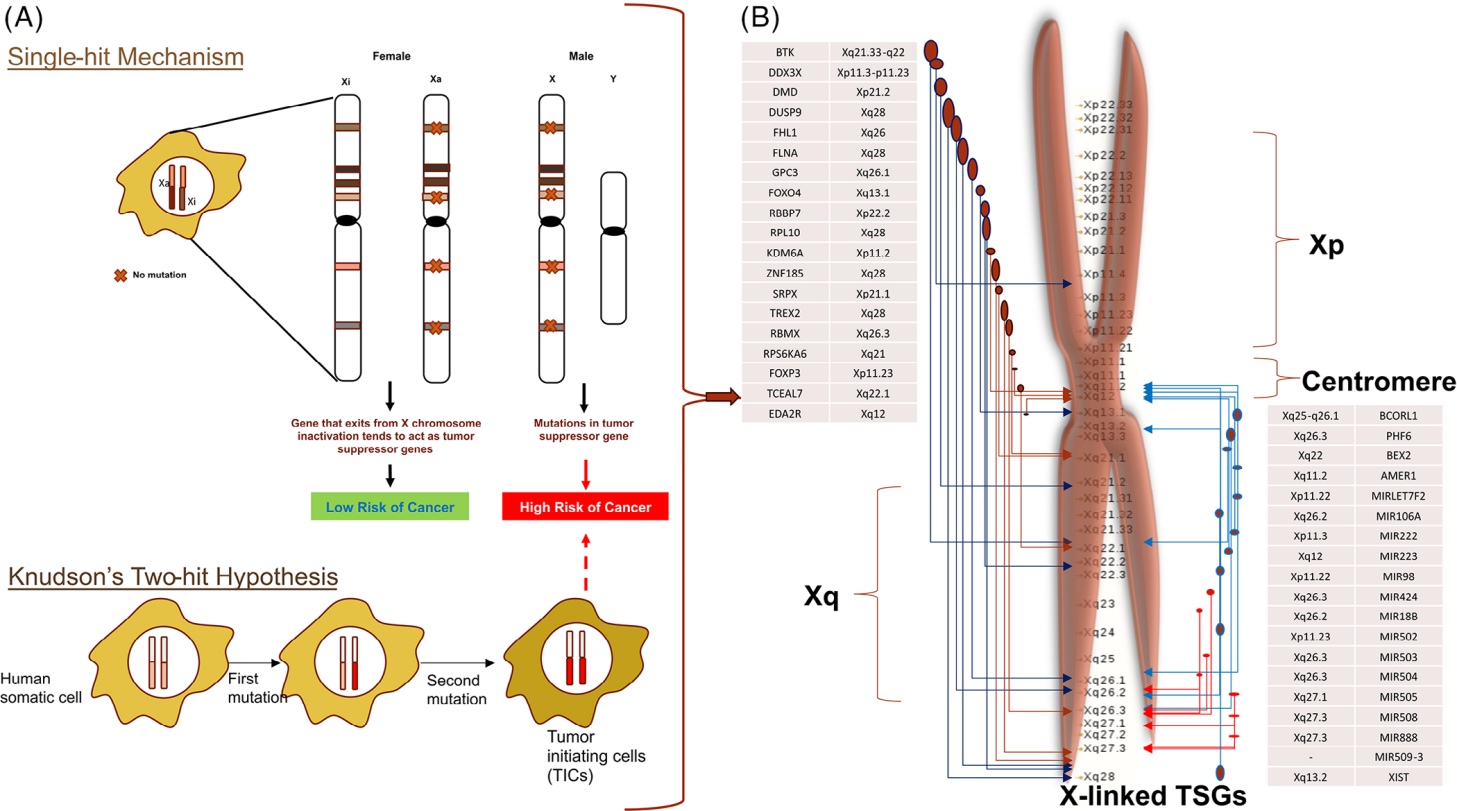
X‐chromosome inactivation and their involvement in cancers: (A) Single‐hit hypothesis (left upper panel) and Knudson's two‐hit hypothesis (left lower panel) and (B) X chromosome showing position of TSGs mainly on its q‐arm (right panel).

Unlike autosomal genes, X‐linked genes are dosage‐compensated to equalize gene dosage between males and females through X‐inactivation (Figure 1) and to achieve grossly similar transcript levels between X‐linked and autosomal genes through upregulation.^22^ After X‐inactivation, female tissues are chimeras of operatively hemizygous cells having active X chromosomes from either parent. This feature has two consequences for TSGs. X‐linked tumor suppressors should be inactivated by one genetic hit, unlike autosomal tumor suppressors that require two.^56^ Single‐hit somatic inactivation and dominant inheritance of X‐linked TSGs are expected.^57^ Although single‐hit inactivation has been proven, no human research has confirmed dominant inheritance. Second, reactivating X‐linked TSGs for cancer treatment may be possible since one allele has not been selected during carcinogenesis.^57^

A mutation in a TSG that occurs in one copy of the gene (heterozygous) has a dominating effect in the family's genetic history. In random cases of the same tumor type that are not related to family history, it is common to find mutations or epigenetic silencing of the TSG in the tumor cells, whereas the normal cells from the same patient do not show these changes.^58^, ^59^ Both instances exhibited tumor cells that had LOH or epigenetic suppression of the normal allele of the identical gene.^58^, ^59^ These discoveries formed the foundation for a comprehensive approach to identify novel TSGs that were not reliant on hereditary cancer cases.^59^ Various experimental approaches have been used to detect tumor‐specific deletions and regions of LOH, such as nucleic acid hybridization using a set of probes for all human genes, screening for single‐nucleotide polymorphisms, and, more recently, quantitative high‐throughput DNA sequencing. The objective of all these discoveries was to identify genomic areas that exhibited frequent deletion or LOH specifically in cancer cells, while being absent in normal cells from the same patients. If the same region exhibited identical effects in tumors from numerous unrelated patients, it could potentially include a TSG.^58^, ^59^

### 3.3 Identification of X‐linked TSGs and TSG databases

Over 20 years ago, the idea of TSGs on the X chromosome was proposed.^24^, ^60^ These potential X‐linked tumor suppressor sites have commonly shown LOH and skewed X chromosomal inactivation (XCI) in breast, ovarian, and prostate malignancies. According to several research studies, up to 40% of cancer samples had LOH of X‐linked genes. Particularly, tumors with germline *BRCA1* mutations commonly have LOH at Xp22.2‐3 of the active X chromosome.^61^ In ovarian cancer, *TP53* LOH is substantially correlated with LOH at Xp25‐26.^62^ These findings imply that these loci may contain TSGs that interact with one another functionally.^21^, ^24^ These X‐linked genes may completely lose their ability to prevent tumor growth as a result of LOH at the functional X chromosome, making people more prone to developing cancer.^21^, ^24^ Extensive LOH at the X chromosome has been linked to higher tumor grade and lymph node metastases.^63^, ^64^, ^65^, ^66^ It is interesting to note that *BRCA1* mutations have been linked to skewed XCI in breast cancer.^67^, ^68^, ^69^ In one study, which included breast and ovarian tumors, it was discovered that 22% of tumors had a decrease in X‐linked gene expression.^70^ Melanoma,^71^ renal‐cell carcinoma,^72^, ^73^ sporadic colorectal carcinoma,^74^ and neuroendocrine tumors^75^, ^76^, ^77^, ^78^, ^79^ are other cancers that have been connected to LOH.

In order to retrieve TSGs and related information, a variety of databases are now available. For instance, the Tumor Associated Gene database (binfo.ncku.edu.tw/TAG/GeneDoc.php), UniProtKB (https://uniprot.org), and the PubMed (https://pubmed.ncbi.nlm.nih.gov/). There exists at least 628 TSGs in mouse, 567 in rat, and 716 in humans (comprising coding genes (*n* = 637), and noncoding (*n* = 79)). Additionally, each TSG's detailed annotations from the database, including information on protein–protein interactions, methylation locations, TF regulations, and cancer mutations, are available for free (http://bioinfo.mc.vanderbilt.edu/TSGene/).^80^ In 2016, Zhao and coauthors created TSGene Version 2.0, which included changes to the contents (such as current literature and the gathering and curation of pan‐cancer genomic data), data kinds (coding DNA and ncRNAs), and accessibility to the content. Protein coding (*n* = 1018) and noncoding (*n* = 199) genes make up the 1217 human TSGs in TSGene 2.0, which can also be viewed free (http://bioinfo.mc.vanderbilt.edu/TSGene). The Cancer Genome Atlas (TCGA)‐derived gene expression and mutation patterns are also provided by TSGene 2.0. There are 38 different types of TSGs on the X chromosome, of which 23 genes code for proteins and the remaining 15 are noncoding genes (https://bioinfo.uth.edu/TSGene/?csrt=13635304675887717882). These TSGS play a role in a variety of cancers, including breast, lung, liver, prostate, and others.^3^

There are a number of text‐mined and regularly updated databases that contains drivers, OCGs, and tumor suppressors gene from many cancers. A comprehensive list of some available databases and resources are available in the Table 2.

**TABLE 2:** A comprehensive list of some available databases and resources for oncogenes (OGs) and tumor suppressor genes (TSGs) along with a short description of the database/resource.

S. No.	Resource/database	Description	References
1	CancerMine (http://bionlp.bcgsc.ca/cancermine)	A literature‐mined resource for cancer drivers, proto‐OCGs, OCGs and TSGs	81
2	DORGE (https://github.com/biocq/DORGE)	Discovery of OCGs and tumoR suppressor genes using Genetic and Epigenetic features	82
3	TAG Database (https://www.binfo.ncku.edu.tw/TAG/)	Well‐characterized database for tumor‐associated genes (TAGs), OCGs, and TSGs to aid cancer research	83
4	TSG Database (http://www.cise.ufl.edu/~yy1/HTML‐TSGDB/Homepage.html)	A web‐based database system with 174 TSGs	84
5	TSGene (https://bioinfo.uth.edu/TSGene1.0/)	Comprising data related to 716 human TSGs (637 coding and 79 noncoding genes)	85
6	COSMIC (https://cancer.sanger.ac.uk/cosmic)	A catalogue of genes with cancer‐causing mutations that explain how gene malfunction causes cancer	86
7	The Human Protein Atlas (https://www.proteinatlas.org/)	An open access resource for human proteins, including oncoproteins and tumor suppressor proteins.	87
8	cBioPortal (https://www.cbioportal.org/)	An open platform for analyzing cancer genomics data, including pan cancer datasets	88

### 3.4 XCI and evolutionary aspects

The phenomenon of disproportionate representation might be partially attributed to the escape of around fifteen percent of X‐linked genes from XCI, a regulatory process that aims to balance the expression levels of X‐linked genes between males and females^89^ (Figure 1). As an example, it has been shown that the *KDM6A*, which is a X‐linked gene evades XCI, although it is commonly identified as being subject to mutations in cases of bladder cancer.^90^, ^91^ In a study conducted by Zuo et al.,^24^ it was found that the *FOXP3* exhibited frequent instances of deletion, mutations, and downregulation in breast cancer samples. The study revealed a high occurrence of DNA methylation‐mediated changes in *FHL1* gene in mouth cancer, highlighting its significant relevance in the context of epigenetics.^92^ Furthermore, previous studies have indicated that the involvement of loci‐specific (specifically on the X chromosome) and global long interspersed nuclear element‐1 (LINE‐1) repetitive sequence is associated with many forms of cancer.^93^, ^94^, ^95^

Genes associated with cancer experience significant evolutionary selection. Researchers have proposed that X‐linked TSGs are not shielded by the Knudson's two‐hit mechanism and are consequently susceptible to negative selection.^57^ Almost all mammalian species showed a decrease in the ratio of TSGs to noncancer genes on their X chromosomes compared with nonmammalian species. They also found that analysis of synteny indicated that there was a decrease in the number of TSGs located on the X chromosome in mammals soon after the XY sex‐determination system evolved.^57^ A model based on phylogeny revealed a greater flow of X chromosome‐to‐autosome relocation for TSGs. The concordance/discordance of chromosomal positions of mammalian TSGs and their orthologs in *Xenopus tropicalis* was evaluated to confirm this in other mammals. In humans, X‐linked TSGs exhibit either a more recent origin or a greater physical size. Through a thorough analysis across multiple types of cancer, it was consistently observed that X‐linked TSGs had a higher occurrence of nonsynonymous somatic mutations. There findings indicate that moving TSGs away from the X chromosome may provide a survival benefit by making it easier to avoid inactivation caused by a single mutation.^57^

The process of X inactivation is reliant on the existence of both the XIC and the X inactive specific transcript (XIST) gene in cis.^96^ However, the extent to which it can be affected by the nature of the chromosomal DNA remains unknown. X inactivation can extend over significant distances at the cytological level, affecting autosomal material in both murine and human X‐autosome translocations. Furthermore, the presence of extra copies of the mouse Xist gene might result in the deactivation of certain autosomal loci. An enigmatic inquiry in XCI pertains to the characteristics of the X‐linked cis‐acting regions that play a crucial role in the attachment and diffusion of Xist across the X chromosome, preceding the suppression of gene activity. There is an increased concentration of LINE‐1 retrotransposons on the X chromosome compared with autosomes. Mary Lyon in 1998 proposed the ‘repeat hypothesis.’ Lyon suggested that these sequences could serve as enhancer elements for the propagation of the inactive signal across the chromosome, leading to effective silencing. However, it has been discovered that Xist does not directly attach to LINE‐1 sequences or form connections with regions that have a high concentration of LINE‐1. Xist utilizes the three‐dimensional structure of the X chromosome to initially expand to locations that are physically close to the Xist gene during the initiation of XCI. Subsequently, it is observed to be more abundant in regions of the chromosome that have a high density of genes but have a lower presence of LINE‐1 sequences.^97^

“Old” X‐linked genes reside on chicken orthologous autosomes 1 and 4, while “new” genes were gained during mammalian X chromosome evolution. XCR genes have orthologues on the marsupial X chromosome, and XAR genes have been introduced since the separation between eutherian mammals and marsupials.^98^ Most single‐copy and multicopy X‐linked genes are shared by humans and mice, while most ampliconic genes are acquired independently.^99^ The marsupial X chromosome is similar to the eutherian mammals' XCR but lacks the XAR, hence XCI is paternally imprinted.^100^, ^101^ Incomplete stochastic XCI may be an old compensatory mechanism based on random monoallelic expression.^102^, ^103^ Most impressively, marsupials lack the XIST gene, but their long ncRNA (lncRNA) RNA‐on‐the‐silent X displays XIST‐like properties.^104^, ^105^ This shows a wide range of silencing mechanisms exist in mammals.

XCI anomalies have also been observed in human cancers.^106^ The lncRNA XIST was formerly considered unnecessary for silencing. New research suggests that blood compartment Xist ablation induces aggressive cancer in female mice.^107^ The authors propose that Xist loss upregulates X‐linked genes, altering genome‐wide homeostatic mechanisms. Mutations in Smchd1, which is linked to cancer in mice, hypomethylate the CpG islands of normally inactivated genes, reactivating X.^108^, ^109^ However, cancer researchers are just beginning to study X‐linked gene epigenetic effects. We know of XCI disruptions but not X upregulation. Further molecular investigations of X upregulation will assist determine its clinical roles. We previously conducted a study to explore the role of X‐linked TSGs and X chromosome–autosome crosstalk and found that although X‐linked TSGs have evaded XCI, these still have a distinct pattern of modified promoter methylation associated with the burden of mutations.^3^ Meiotic sex chromosome inactivation (MSCI) affects most or all X chromosomal protein‐coding genes in mice, according to transcriptomics study conducted by Royo et al.^110^ Whether X‐linked ncRNAs behave similarly is unknown. microRNA (miRNA) genes are abundant on the X chromosome and many are testis‐biased. Importantly, they found that pachytene spermatocytes express significant quantities of X‐linked miRNAs, suggesting that these genes may escape MSCI and play a role in XY‐silencing. Some of the most intriguing stories of recent years include lncRNAs as master regulators. Mammals’ first epigenetic lncRNAs were found in genomic imprinting and XCI investigations. Such lncRNAs may recruit chromatin‐modifying complexes to suppress or activate genes in allelically controlled clusters. Lee^111^ suggests that allelic regulation is ideal for lncRNAs. lncRNA's anchoring and fast turnover make them good allelic markers. The RNA polymerase II transcription complex links these transcripts to the synthesis site, making them allele‐specific tags. Xist and RepA RNA reveal that lengthy transcripts can cotranscriptionally acquire chromatin complexes while fixed to the transcription site.^112^ Bridge proteins like YY1 help tether Xist RNA.^113^ Collectively, XCI, genomic imprinting, and lncRNA affect public health greatly. To manage X‐linked disease and disorders, few preventive, diagnostic, and therapeutic techniques have addressed imprinted gene and Xic regulatory factors.

### 3.5 Targeted therapies for tumors through restoration of activity of TSGs

Restoring inactivated tumor suppressors is a major cancer treatment hurdle. Female cancer cells commonly have heterozygous FOXP3 and WTX deletions or mutations. Thus, reactivating the X‐inactivated TSG for cancer treatment is intriguing. The mechanism of X‐inactivation induction is well recognized, but its maintenance in normal cells is not (Figure 1). Any logical attempt to reactivating X‐linked TSGs faces significant challenges. We found a fortuitous observation that implies this may be possible. In summary, anisomycin, a cellular stress inducer, activated c‐Jun and ATF2 heterodimers to stimulate FOXP3 expression in mouse and human breast cancer cell lines.^114^ This stimulation boosted cancer cell death and decreased mice mammary tumor development.^114^

First and foremost is the risk of reactivating X‐linked genes. Xist deletion in the paternal X chromosome causes growth retardation and embryonic lethality in female mice.^115^ The effects of partial or full X‐reactivation in mature animals are unclear. Global investigation of X‐inactivation reveals that 10% of X‐linked genes display varied inactivation patterns and are expressed from certain “inactive” X chromosomes, in addition to the clusters of genes that generally escape inactivation.^116^ This shows humans tolerate differences in X‐inactivation of at least 10% of genes. However, since pharmacological reactivation of X‐linked genes has not been performed, caution and openness are advised.

Selectively reactivating X‐linked tumor suppressors in cancer would lessen negative effects. As described below, many tumors have partially disassembled their X‐inactivation machinery, making this conceivable. Richardson et al.^69^ found that basal‐like tumors, most of which have BRCA1 mutations and poor prognoses, have nonheterochromatinised X chromosomes. This confirms a prior observation that heterochromatinized X chromosome deletion is related with poor prognosis. H3mK27 and Xist, indicators of inactive X chromosomes, are decreased or missing, and many genes lose DNA methylation.^69^ Interestingly, only one example showed bi‐allelic expression of a typically X‐inactivated gene. Array research shows that just 3% of 1200 X chromosomal genes are overexpressed.^69^ X‐linked genes may not be reactivated since most of the overexpressed genes are located at or near Xp22 and Xq26‐28 loci, which are rich in genes that ordinarily survive X‐inactivation.^69^ Xist, DNA methylation, and histone hypoacetylation work together to inactivate X‐linked genes (Figure 1), therefore losing Xist and DNA methylation should lessen the steps needed to reactivate them.^117^, ^118^ Because methylation is differentially changed among genes, reactivation may not be effective for all genes. This may allow selective reactivation and reduce negative effects.^69^

Down the line, it is also reasonable to explore if reactivating X‐linked tumor suppressors is therapeutic. Elegant investigations showed that tumors can become “addicted” to Trp53 (p53) depletion.^119^ Ectopic FOXP3 expression inhibits almost all tumor cell types despite the lack of analogous research with the two known X‐linked tumor suppressors.^120^, ^121^, ^122^, ^123^ Recent anisomycin studies have shown that FOXP3 expression in mice and human tumor cells is therapeutic.^114^ Carcinogenesis often involves TSG dysfunction. TSGs lose function due to decreased miRNA expression, a marker of malignancy.^124^ Epigenetic silencing of TSGs is one of the universal abnormalities found in all malignancies. TSG silencing may start the oncogenic process.^125^

## 4 X CHROMOSOME–AUTOSOME CROSSTALK

In mammals, the X chromosome is unique. Although, the sex‐determining chromosome is the Y chromosome. In males (XY), the X chromosome is present in single copy numbers leading to the distinctive pattern of X‐linked inheritance that has allowed the allocation of many genes.^116^ However, the X chromosome constitutes more or less five‐hitter of the human genome sequences. The duplication of this genetic material in females required a dosage‐compensation system to silence transcriptionally one X‐chromosome copy. One among the pairs of the X chromosomes is completely deactivated during initial stages of fetal development. The participation of genes on X chromosome in cancers has typically been analyzed by their loss or activation of genes as a result of dynamic changes in chromosomes. XCI has decelerated the involvement of this chromosome in cancer since X chromosomes that are implicated in aneuploidies and rearrangements is also either active or inactive.^126^

Several theories related to dosage compensation are known to regulate the X chromosome gene expression level, which assures equal gene expression levels between the X chromosome and autosomes, and expression levels between the sexes. The sex chromosomes are differentially concerned with a comparably massive, gene‐rich X chromosome and a little, gene‐poor Y chromosome that degenerated due to suppressed recombination to avoid abnormal transfer of the male determinant. In mammals, dosage compensation is accomplished by higher expression levels of dosage‐sensitive X‐linked genes (that is, X upregulation) in both sexes and by silencing or inactivation of one copy of X chromosome (X‐linked genes will be classified on the basis of their copy number, evolutionary history, mode of X upregulation, and XCI status. Copy number: X‐linked genes are classified as single‐copy, multicopy (that means, genes with ≥2 copies, however, do not seem to be not located in ampliconic regions (that is, genes present in segmental duplications of >10 kb in length that share >99% nucleotide identity), evolutionary history: “Old” Xlinked genes are chicken orthologous autosomes 1 and 4, whereas “new” X‐linked genes square measure people who are noninheritable throughout the evolution of the X chromosome, mode of X upregulation: increased expression levels of genes on the single active X chromosome to balance expression with the autosomes, XCI status: silencing of one X chromosome. Usually, the gene that escapes XCI has been defined as one that shows ≥10% expression from the inactive X allomorph compared with the active X allelomorph.^116^ Escape genes will be exclusively expressed from the inactive X chromosome, for instance, XIST.^127^ Mutations, deletions, and copy number variations of escape genes evoked abnormal phenotypes, and varied diseases as well as cancer. For instance, mutations in the escape gene *KDM6A* are concerned in medulloblastoma, prostate cancer, and renal carcinoma.^128^, ^129^ *KDM6A* mutations cause Kabuki syndrome, which could be an inborn syndrome represented by skeletal abnormalities, growth retardation, and mild to severe intellectual disability.^130^, ^131^, ^132^ Curiously, heterozygous females are affected to various degrees, most likely as a result of low expression levels from the conventional allelomorph in cells within which it is on the inactive X chromosome.^133^, ^134^ Another escape gene associated with an intellectual disability is *KDM5C*, which is important for neural cell development.^135^, ^136^

The X‐linked TSGs are involved in multiple types of cancer. X‐linked TSGs, directly and indirectly, interact with sex chromosomes and autosomes through genetic, physical, and biological interactions, resulting, which can lead to various types of diseases, including cancer. Ras Association Domain Family Member 1 (*RASSF1C*) located on chromosome 3, which interacts with Bax, Caspase 3, and *SRPX* gene could promote cell migration and downregulate apoptosis.^137^ The *BRCA2* gene associated with *TREX‐2* complex subunit PCID and DSS1 that are implicated in messenger ribonucleoprotein (mRNP) biogenesis and export and maintenance of the genome stability.^138^ These associations use to indicate that R‐loop is the major cause of replication‐related stress and cellular instability.^139^ Ribosomal protein S6 kinase, 90 kDa, and polypeptide 6 gene (*RPS6KA6*) shown interaction with the *P53* gene and modulate its function as well as the Ras‐MAPK pathway, epigenetic pathways, and gene expression programs, which cause colorectal cancer.^140^ *WTX* gene also known as *AMER1* physically interacts with *APC*, which is a TSG involved in colorectal cancer.^141^ Tumor suppressor *DUSP9* gene located on the X chromosome regulates cell proliferation and predicted altered gene profiling in hepatocellular carcinoma (HCC) biology.^142^

To find the genes that function as TSGs, a number of investigations have been done in the past.^1^, ^3^, ^4^, ^24^, ^89^ The protein‐coding gene for *BTK* functions as a TSG throughout the mouse's B‐cell development. Regardless of its enzymatic activity, the gene acts as an adapter macromolecule in pre‐B cells to restrict or limit tumor growth.^143^ The ZNF185 protein, along with other X‐linked genes, belongs to a family of LIM‐domain‐containing proteins. By using a cell growth and soft agar colony formation assay, San and coauthors investigated the role of *ZNF185* in the initiation and growth of prostate cancer. They found that, when the *ZNF185* gene was overexpressed through a transfection experiment, the gene had a major antitumor effect on the progression of prostate cancer cells and colony formation in soft agar.

### 4.1 OCGs and TSGs

Zhu et al.^144^ conducted an interesting study, wherein they investigated the OCG and TSG networks to offer new insights into their relationship in the local network organization and environment. They created a TSG‐OCG network using human PPI networks, consisting of 50 TSG proteins and 50 OCG proteins.^144^ The TSG‐OCG network comprised 106 nodes and 303 edges, as shown in Figure 6. Out of the 106 nodes, 48 were TSG proteins, representing 96% of all TSG proteins; 49 were OCG proteins, representing 98% of all OCG proteins; and nine were linkers. The network composition showed that the TSG‐OCG network primarily comprised TSG and OCG proteins. Out of the 303 edges, 89 connections were found between 42 TSG proteins, 51 connections between 36 OCGs, 117 connections among 71 proteins (38 TSGs and 33 OCGs), and 46 connections between nine linkers and 15 TSGs or 26 OCGs. Therefore, 257 connections (84.8%) were found between TSGs and OCGs, indicating a strong interconnection between the proteins of TSGs and OCGs in protein–protein interaction networks. The proportion of linkages between the 38 TSGs and 33 OCGs (38.7%) was higher than the interactions among the TSGs (29.5%) and OCGs (16.9%) individually. The majority of the TSGs (38, 79%) shared at least one edge with OCGs. Most of the OCGs (67%) shared at least one edge with TSGs.

TSG and OCG proteins exhibited larger degrees, higher betweenness, lower clustering coefficients, and shorter shortest‐path distances compared with target proteins, essential proteins, and other proteins. Furthermore, the TSG and OCG proteins showed no significant difference in terms of network topological features. Both TSG and OCG proteins exhibited a higher frequency of direct interactions with target proteins.^144^

### 4.2 Cellular signaling circuits connected to X‐linked TSGs

**FIGURE 2:**
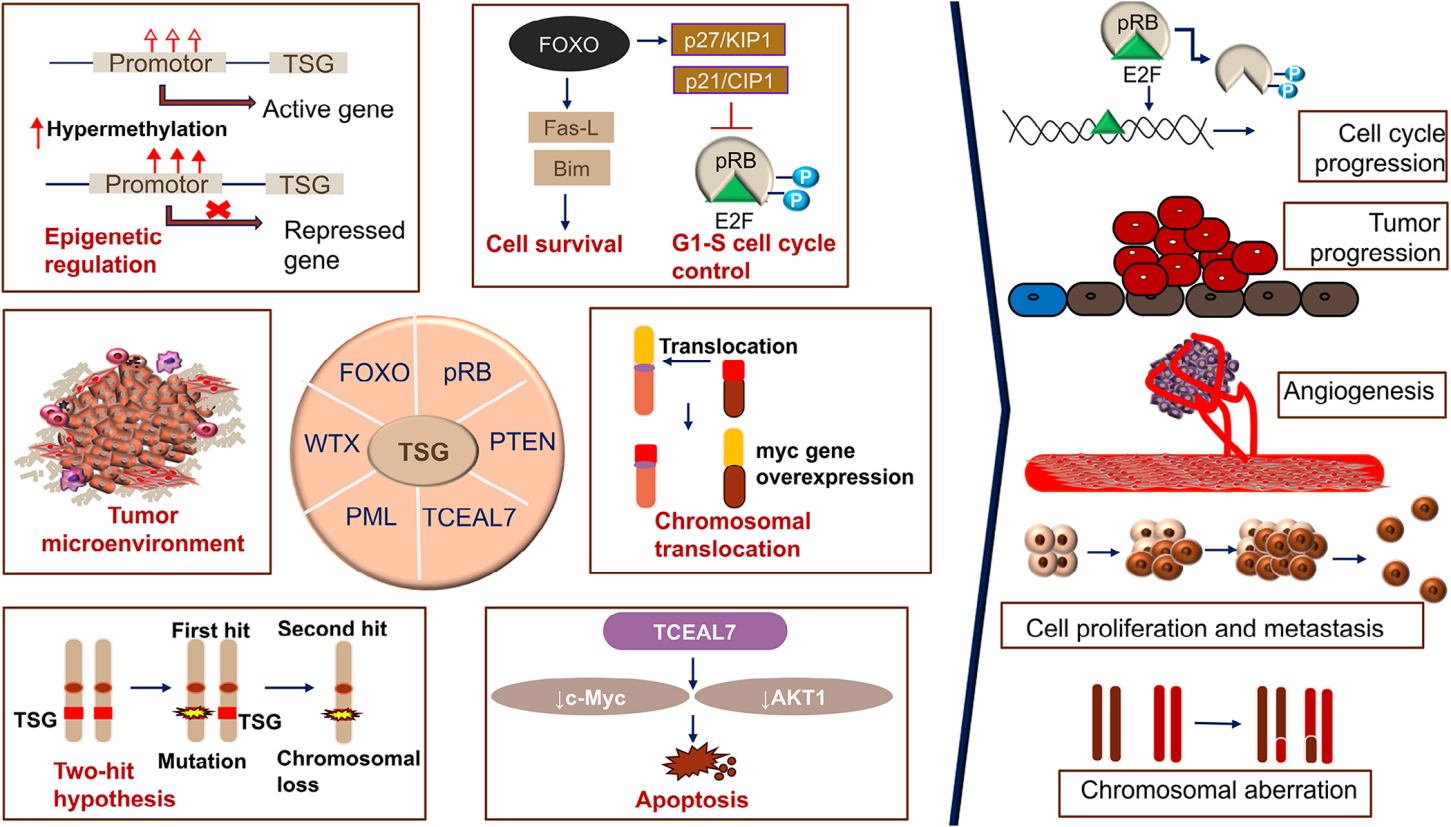
Cell signaling circuitry related to X‐linked tumor suppressor genes. TSGs encoding variety of proteins that help to regulate cellular growth are described. Genes playing a role in the control of cell surface receptors for cytokines, growth factors, signal transduction molecules, and transcription factors, epigenetic regulators, regulators of the cell cycle, and regulators of apoptosis in different cancers are shown.

TSGs can encode a variety of proteins that help regulate cellular growth.^145^ According to Ref. 146, these genes play a role in the control of cell surface receptors for cytokines, growth factors, signal transduction molecules, and TFs, as well as epigenetic regulators, regulators of the cell cycle, and regulators of apoptosis in different cancers (Figure 2). According to Ref. 147, TSGs are typically thought of as negative regulators of cell development that are effective upon invasive and metastatic ability. Additionally, TSGs and proto‐OCGs are crucial for the growth of myeloid cells. Acute myeloid leukemia (AML) can be caused by a mutation in this type of cell.^148^

By targeting the important protumor/tumor suppressors in cancer pathways, cells escape growth control.^1^, ^3^, ^4^ Through the conventional evolution of cell and tissue repair, these pathways have implicated both growth promoting and negative signals regarding biological role, cell growth, cell‐cycle progression, and microenvironment. Since the RB pathway and the p53 pathway are both usually targeted at the tumor's origin, the mutation that occurs in each pathway is dependent on the type of tumor. For instance, the phosphoinositide 3‐kinase (PI3K)/AKT pathway is likely the target of *FOXO4* X‐linked TSG during the tumorigenic process.^149^ The RAS–methyl‐ethyl‐ketone (MEK)–extracellular signal‐regulated kinases (ERK), IKK, and AMPK pathways, as well as others, are connected to FOXOs. Forkhead/winged‐helix TF family members include the X‐linked TSG FOXP3. In men, autoimmune disorders brought on by germline mutations can be fatal. The majority of the tumors were discovered to be mammary carcinomas with overexpressed HER‐2/ErbB2 and inactivated wild‐type *FOXP3* alleles. The HER‐2/ErbB2 promoter was bound by *FOXP3* and inhibited. Regardless of the presence or absence of HER‐2 amplification, deletion, functionally important somatic mutations, and downregulation of the *FOXP3* gene were frequently observed in human breast cancer samples and significantly linked with HER‐2/ErbB2 overexpression. A percentage of Wilms tumors have the specific sequence mutation WTX/AMER1. On a molecular level, *WTX* has been associated with both normal and cancerous development by acting as potential contender of the Wnt/β‐catenin pathway in fish and mammals.^141^ In ovarian surface epithelial cells, transcription elongation factor A‐like 7 (*TCEAL7*) increased growth (adherent independent) and changed Myc functionalities. The results of protein/DNA array analysis show that nuclear factor NF‐B binds to its target DNA sequence nearly twice as often when *TCEAL7* is downregulated. Rattan et al.^150^ noted that *TCEAL7* downregulates the NF‐κB mediated gene expression by regulating the binding of NF‐κB on the promoters of its target genes. This happens through repressing the activation of NF‐κB in *TCEAL7* downregulated clones, IOSE‐523, and in other ovarian cancer cell lines (OVCAR8, SKOV3ip, and DOV13), and inhibiting p65 transcriptional activity, by itself or by tumor necrosis factor‐α. As a result, the study suggested that *TCEAL7* may have a unique role in negative regulation.^150^ Furthermore, *EDA2R* was discovered to be a P53 target gene by Tanikawa et al. in 2009. *EDA2R* may function as a growth suppressor and contribute to the emergence of colorectal cancer. According to victimization RNA‐seq data, TP53 directly targets EDA2R.^151^ *BEX2* and miRNA‐370 are essential for the development of HCC. *BEX2* and miRNA‐370 both had high expressions in the HCC cell line. MiRNA‐370 inhibited the MAPK/JNK signaling pathway by targeting *BEX2*, which had an antitumor effect on the progression of HCC.^23^

### 4.3 Regulation of X‐linked TSGs

TSGs typically inhibit a variety of signaling pathways, including biological, molecular, and cellular pathways. Different types of cancer are caused by changes to TSGs caused by a variety of causes. TSG expressions are regulated by a number of variables, including mutation, methylation, TFs, noncoding genes, and SINES/LINES (repetitive portions). The modulation of gene expression plays a critical role in deciding the ultimate destiny of a cell. The regulation of gene expression encompasses multiple stages, with the commencement of transcription being the most significant regulatory point. Cells utilize the transcriptional regulatory mechanism to facilitate processes such as cellular division, growth, and proliferation. The process of transcriptional regulation is carried out by a complex network of TFs, which are believed to be conserved throughout the course of evolution. Transcriptional regulation plays a significant role in various cellular processes, encompassing both physiological and pathological contexts. It influences normal development as well as the advancement of malignancies.^152^, ^153^, ^154^ The regulation of gene expression is a critical process that involves the activation and suppression of gene activity. This regulation mostly occurs through the utilization of gene promoters, which are nucleotide sequences located within a range of 100 bp downstream to 1000 bp upstream of the start site of gene transcription (TSS).^155^ These gene promoters possess specific regulatory sequences that control the transcriptional activity of genes. The promoter region is comprised of three distinct components, with the first component being referred to as the Core Promoter. The core promoter, which is located around 200 bp upstream of the TSS, encompasses the TATA, GC, and CAAT box. The former and initiator (INR) sequence motifs hold significant importance due to their immediate recognition by general TFs and their role in facilitating the assembly of RNA polymerase.^156^, ^157^ In addition to the TATA box and INR, the GC box and CAAT box have been identified as prominent promoter elements. It has been established that various RNA polymerases and a substantial number of TFs interact with these elements in a sequence‐specific manner.^158^, ^159^ TFs serve as master regulators, exerting control over numerous cellular processes and influencing the fate of cells, including specific pathways such as immune responses. The presence of chaos in TFs or TF binding sites eventually have pathogenic role in development of numerous disorders, including cancer.

A number of genes influence how cells function and may even regulate the expression of other genes by binding to their promoter region.^1^, ^3^ These genes are known as TFs, or nucleotide‐specific DNA‐binding factors. Nucleotide‐specific DNA‐binding factors that bind to specific sequences found in the promoter, enhancer, or other regulatory regions of DNA control the transcription process. According to Johnston et al., TFs are strongly associated with the development and progression of cancer. For instance, SP/KLFs bind with OCGs and tumor suppressors and have the potential to be oncogenic and alter the expression of these genes. Other members of this class of TFs have also been found in a variety of cancer types. TFs are central players in regulating the gene expression and production of protumor proteins. Insightful ideas for the study and treatment of cancer were generated by results that identified TFs that may result in differential expression of genes in cellular pathways of cancer. According to Refs. 152, 153, 154, TFs are one of the major variables that are critical to normal cellular physiological and pathological consequences as well as the growth of cancer. Increased cell growth, cell division, and expression of genes involved in the Wnt signaling pathway and the MAPK signaling pathway are caused by the regulation of TF *NFE2L2* on genes.^160^, ^161^, ^162^ Additionally, genes associated with cell adhesion molecules and cytokine‐cytokine receptor interaction pathways may influence immune system pathways that have a high correlation with TF *XBP1*. Additionally, the CGC dataset analysis supports the strong link that these two TFs have with genes that exhibit comparable patterns. As a TF, early growth response factor 1 (*EGR1*) primarily affects fibrosis, immunological responses, and the damage of tissue. Recent research has revealed that *EGR1* is closely associated to the development and spread of cancer and may participate in tumor angiogenesis, invasion, and metastasis. Although *EGR1* controls these processes, the precise method by which it does so is still unknown.^163^, ^164^

**FIGURE 3:**
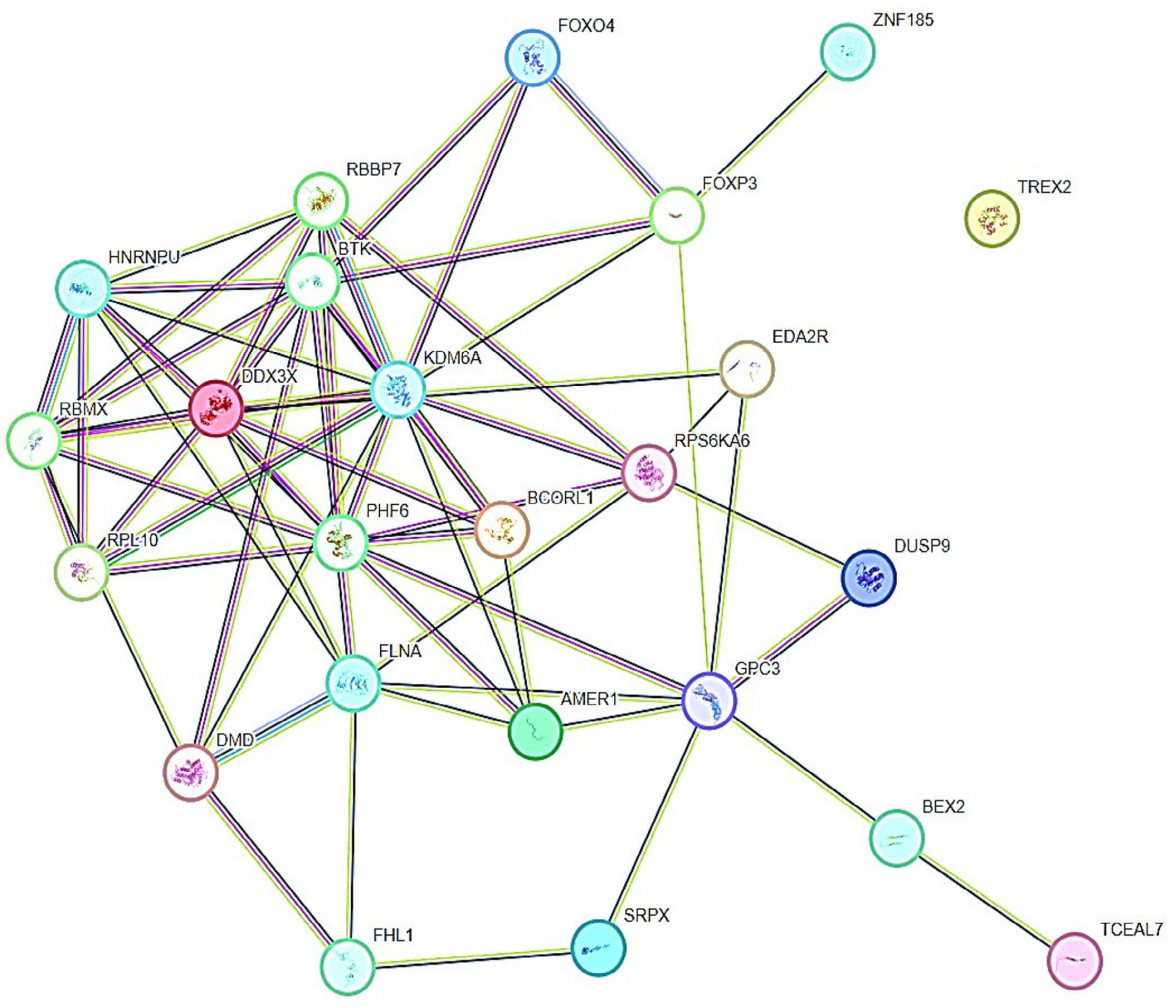
STRING‐based gene interaction maps and network circuitry. Tumor suppressor genes, including X‐linked tumor suppressor genes having molecular interactions with other target genes or proteins through physical and functional associations are shown.

**FIGURE 4:**
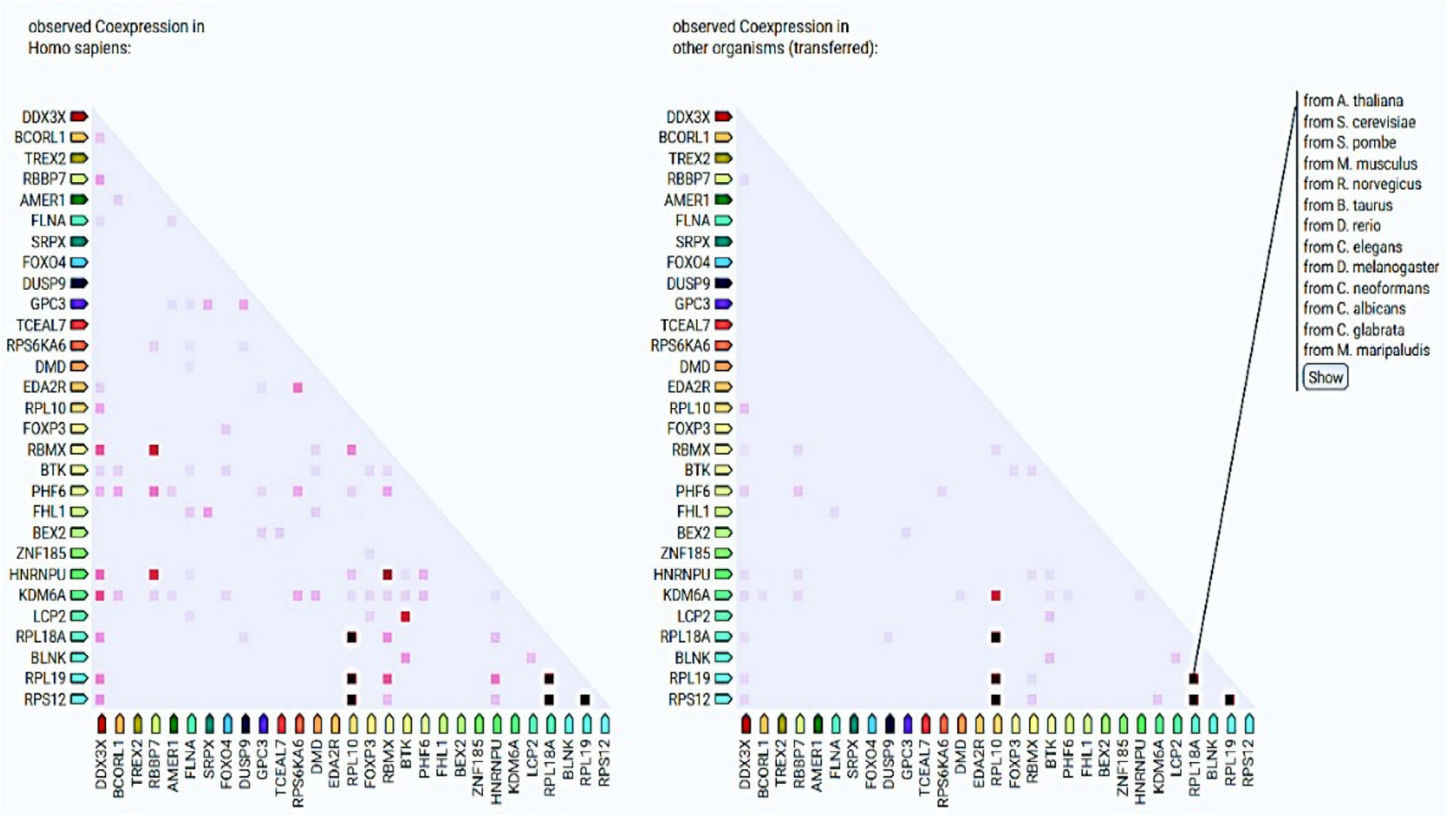
A heatmap representation of the coexpression scores of X‐linked TSGs based on RNA expression patterns and on protein c‐regulation provided by ProteomeHD.

TSGs, including X‐linked TSGs establish specific molecular interactions with other target genes or proteins through physical and functional associations (Figure 3). TSGs interact with a number of genes or proteins through a spectrum of biological processes and phenomenon related to cancer, especially epigenetic regulation of different cancers. The gene *UTX* (also known as *KDM6A*) encodes a histone H3K27 demethylase and is a tumor suppressor frequently altered in human cancers.^165^ *UTX*’s molecular activity is uncertain because its demethylase activity is generally insufficient for tumor suppression and developmental regulation.^166^, ^167^, ^168^ Forkhead box P (FOXP) TFs—*FOXP1*, *FOXP2*, *FOXP3*, and *FOXP4*—are involved in embryonic development, immune disorders, and cancer progression, but *FOXP3*’s targeting of CD4 + CD25+ regulatory T (Treg) cells and its dual role as an OCG or tumor suppressor in cancers are unclear and controversial.^169^ *FOXP3* interacts with *FOXP4*, *ZNF185*, *GPC3*, *BTK*, and *KDM6A* (Figure 3). PPI network have shown that *GPC3* upregulates in *TP53*/*EGFR* double mutant.^170^ RBBP7, a ubiquitously expressed nuclear protein from the polycomb repressive complex 2, has HMT activity for histone H3 Lys 9 (K9) and Lys 27 (K27).^171^ Additionally, NKX6.1 directly represses vimentin by interacting with the *RBBP7* corepressor, which raises H3K27me3 levels. Li et al.^172^ found that *NKX6.1* interacts with *BAF155* and *RBBP7* to stimulate epithelial gene expression and suppress mesenchymal gene expression at the transcriptional level. Besides this, TSGs to very less extent coexpress with other TSGs (Figure 4). *RPL10* coexpresses with *RPL12*, *RPL18A*, and *RPL19*. *RBBP7* coexpresses with *RBMX* and *HNRNPU* (Figure 4). We believe that physical and functional associations of TSGs with other genes or proteins need more evidence from experimental endeavors.

### 4.4 Dysregulation of X‐linked TSGs

TSGs are often downregulated in cancer, dysregulating their pathways. The continuum model of tumor suppression argues that even minor changes in TSG expression, such as epigenetic modifications or copy number adjustments, can cause gene function loss and phenotype effects. Some malignancies dysregulate X‐inactivation maintenance, although worldwide reactivation has not been described. The peculiar condition of X‐inactivation in cancer makes selective reactivation of X‐linked TSGs possible. The *TP53* mutation's impairment of XCI mechanisms prompted our in‐depth research of BRCA cohort X‐chromosome integrity. Researchers have found TP53 mutation enrichment in tumors with substantial Xi deletions and big Xa amplifications when investigating dysregulated XCI.^173^ The data suggested that WT‐p53 suppresses altered X‐chromosome CN and major X deletion and duplication events. These novel findings on WT‐p53 and X‐chromosome ploidy in adult female breast tissues support its role in reducing aneuploidy.^174^

Defective X‐linked TSGs’ regulation also results in failure of the cell cycle regulation. Cell cycle progression is restricted by TSGs. Genetic changes cause their deactivation and loss of cell division control. In vitro transformation experiments or more complex in vivo animal models can determine how both types of genes affect tumor formation. These experiments will improve our understanding of cancer genetics, cell cycle regulation, and cancer treatment.

### 4.5 Changes in epigenetics landscape of TSGs

**FIGURE 5:**
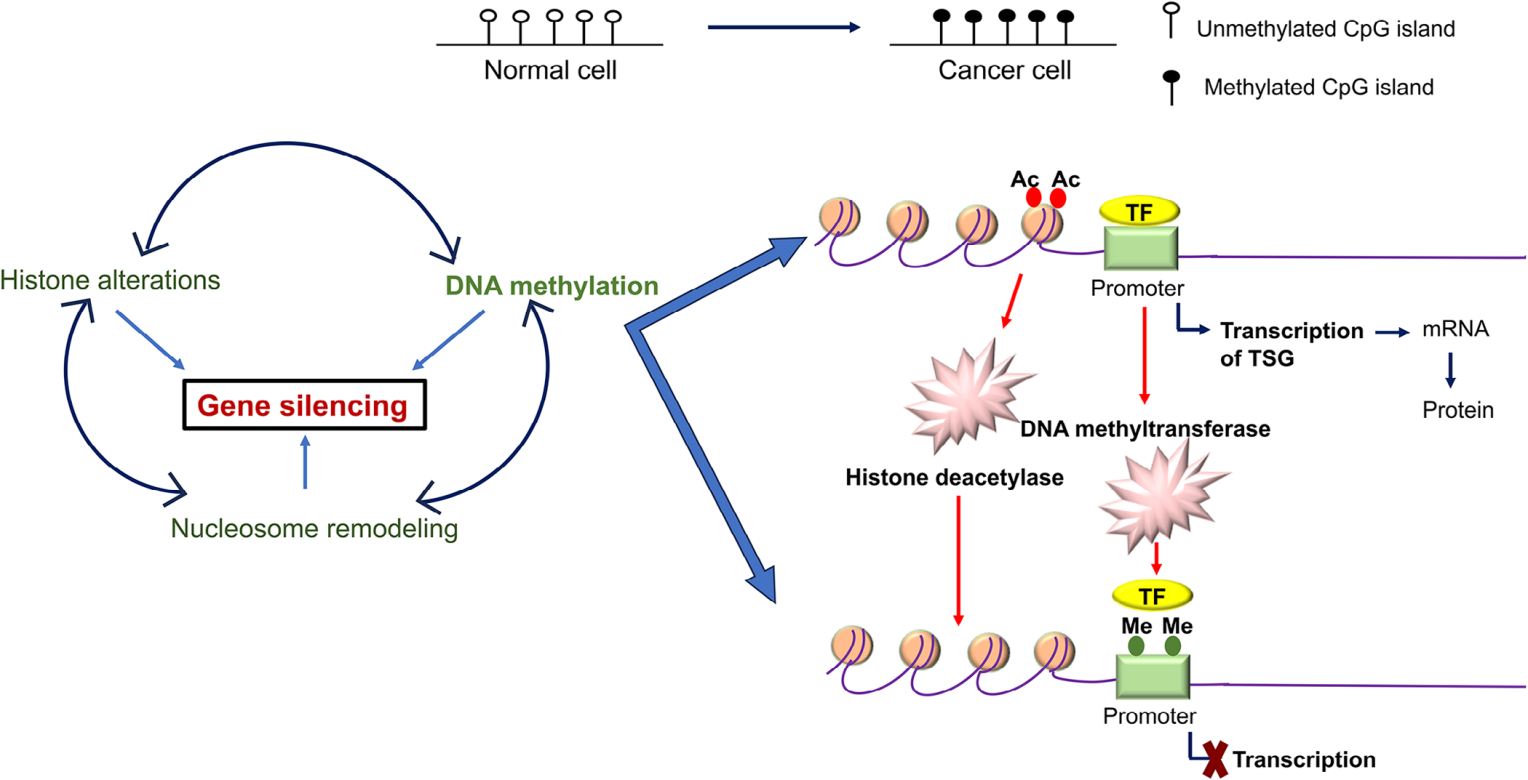
A comprehensive figure depicts the epigenetic silencing of tumor suppressor genes through various alterations including DNA methylation, nucleosome remodeling, and histone alterations. The CpG island in the promoter region methylates and induces the normal cell to metastasis contributing in the development of cancer cells by inactivating the tumor suppressor gene transcription.

To the best of my knowledge, only a few years after the OCG mutation in the H‐Ras in a human primary tumor was revealed, the *RB* gene was the first to exhibit CpG methylation of a TSGs in malignancy in year 1989.^175^ The TSG p16INK4a was typically inactivated by hypermethylation caused by CpG island.^176^, ^177^, ^178^ Since then, the number of potential genes with purportedly abnormal CpG island methylation has increased dramatically,^179^ the hypothesis that, in comparison with healthy tissue, the genome of cancerous cells experiences a decrease in the amount of 5‐methylcytosine.^179^ Genes involved in cell cycle (p16INK4a, p15INK4b, Rb, p14ARF), DNA repair (*BRCA1*, *MLH1*, *MGMT*), carcinogen‐metabolism (*GSTP1*), cell‐adherence (*CDH1*, *CDH13*), and apoptosis (*DAPK*, *TMS1*) are all impacted by the promoter hypermethylation due to the presence of CpG island.^180^ Esteller and colleagues' seminal study from 2001 was the first to demonstrate high‐frequency promoter methylation in a variety of cancer types. They suggested that aberrant DNA methylation of gene promoters may serve as markers for the sensitive detection of practically all cancer types and showed that common abnormalities in DNA methylation may be a major hallmark of oncogenesis (Figure 5). Twelve cancer‐associated genes, which cover 15 major tumor types and were selected from over 600 original tumor samples in the study, were utilized to demonstrate promoter hypermethylation using a candidate gene approach. The profile of promoter hypermethylation, which is unique to each type of tumor, suggested that changes in DNA methylation are ambient, but the overall impact on numerous signaling pathways that are distinctive to the tumor.^181^

The most common epigenetic alterations that could occur in cancer is the aberrant DNA hyper/hypomethylation of TSGs, and alterations in the patterns of histone proteins and their binding to the chromatin (Figure 5). As a result, these changes have received much research. Due to the fact that some miRNAs are negatively regulated in cancer and function as real TSGs, this information junction supports the idea that miRNAs can be suppressed by epigenetic changes. miRNAs such as has‐miR‐127, has‐miR‐124a, has‐miR‐124a‐2, and has‐miR‐124a‐3 are miRNAs that are related to tumors and are rendered inactive by methylation in tumor cells.^182^, ^183^, ^184^, ^185^ Methylation of DNA and gene silencing are shown to be coupled by proteins having methyl‐CpG binding domains (MBD). This biological characteristic shows that TSGs that are hypermethylated at their promoter regions in cancer cells are inactivated by MBD proteins. However, it has been shown that MBD proteins are present in the most hypermethylated promoters of many genes, whereas unmethylated promoters are often devoid of MBD proteins with the exception of MBD1. The idea that the interaction of MBDs with methylated promoters is methylation‐dependent is supported by the observation that treatment of cancer cells with the demethylating drug 5‐aza‐2′‐deoxycytidine results in promoter area hypomethylation, MBD release, and gene re‐expression. Alternative promoters seem to be less exclusive than many promoter sequences, which are quite selective in engaging a certain set of MBD. According to a study by Lopez‐Serra and colleagues, MBDs show a high affinity for the in vivo binding of TSGs with hypermethylated promoter CpG islands, and their occupancy profiles vary depending on the gene and type of tumor,^186^, ^187^ methylation inactivated a new candidate TSG *TCEAL7* in ovarian cancer. Methylation of a CpG site inside the promoter is correlated with negative regulation of *TCEAL7* gene in both tumors and tumor cell lines. Promoter activity is suppressed in vitro when the CpG site is methylated, but the inhibition is lessened when the *Sma*I site is selectively demethylated. Last but not least, *TCEAL7* re‐expression in cancerous cell lines promotes cell apoptosis and decreases colony formation efficiency. The fact that *TCEAL7*, a death/apoptosis regulating protein, has been found to frequently be inactivated in ovarian malignancies among these data suggests that it may function as a tumor suppressor in this cancer type.^188^

### 4.6 Functional participation of noncoding regions in regulation of TSGs

**FIGURE 6:**
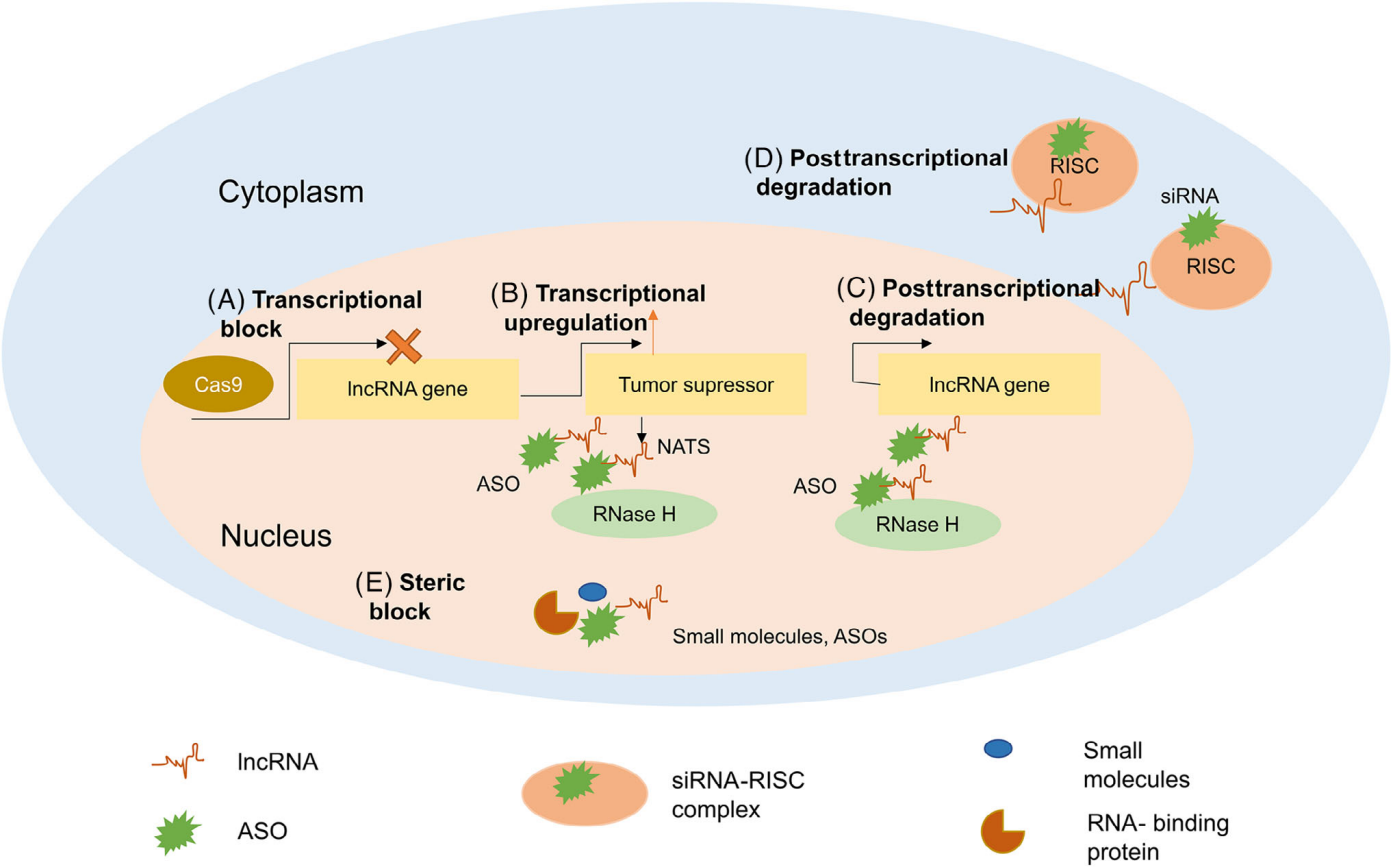
The schematic representation summarizes different approaches to target lncRNAs in the cytoplasm and nucleus. (A) Transcriptional inhibition can be attained by CRISPR/Cas9 to delete regions of interest in the loci of lncRNA. (B) Transcriptional upregulation of tumor suppressors can be attained by knocking down of the corresponding natural antisense transcripts (NATs). (C) Antisense oligonucleotides (ASOs) can posttranscriptionally knock down lncRNAs that are overexpressed in cancers. (D) Posttranscriptional silencing can be attained by siRNAs targeting lncRNAs. siRNAs stimulate dicer activity and recruit the RISC complex (RNA‐induced silencing complex) to posttranscriptionally degrade target RNAs. (E) Steric inhibition of lncRNA–protein interactions can be achieved using small molecules, or modified ASOs that cannot stimulate an RNA degradation pathway.

**FIGURE 7:**
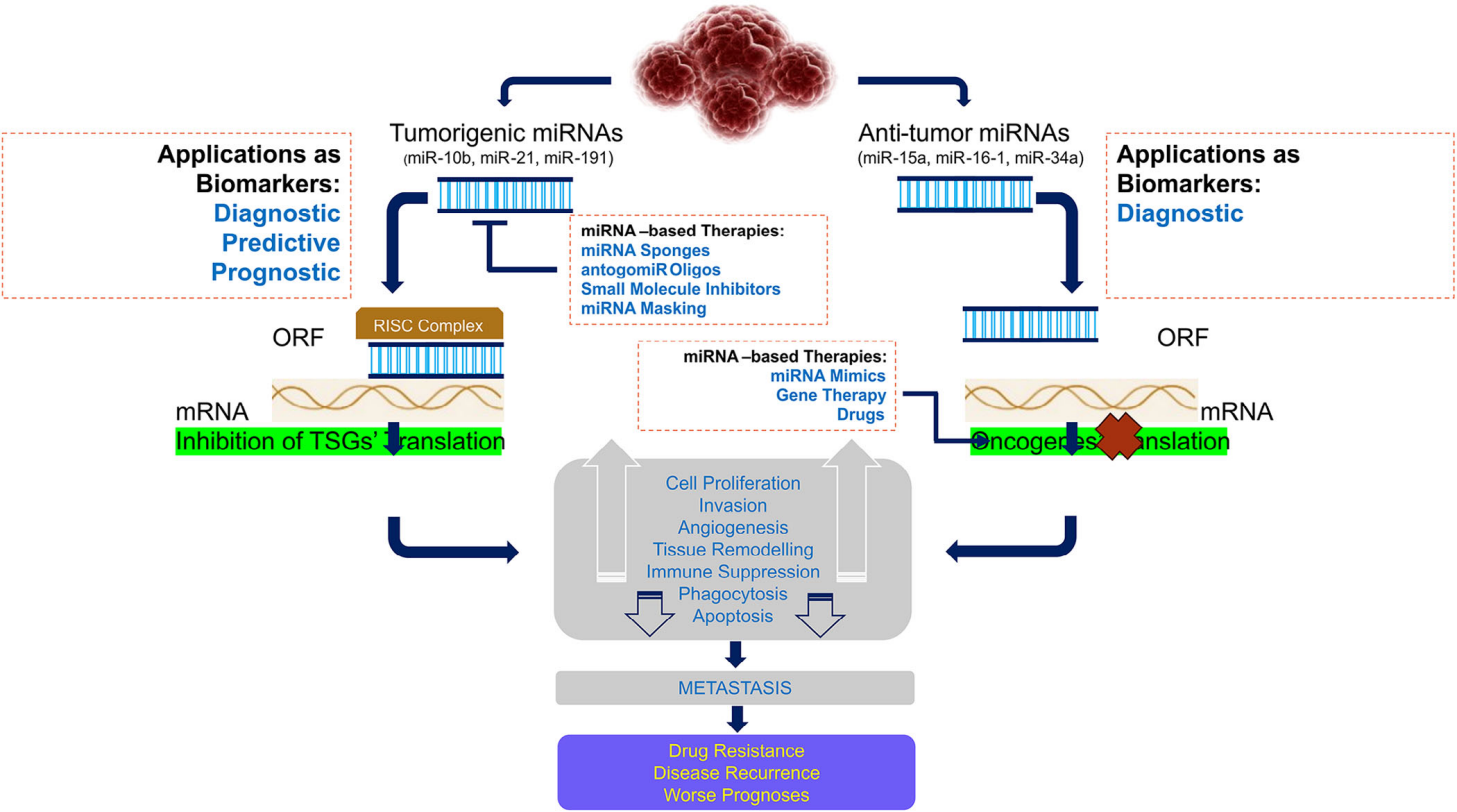
Functional implications of tumorigenic miRNAs (onco‐miRs) and antitumor miRNAs.

**FIGURE 8:**
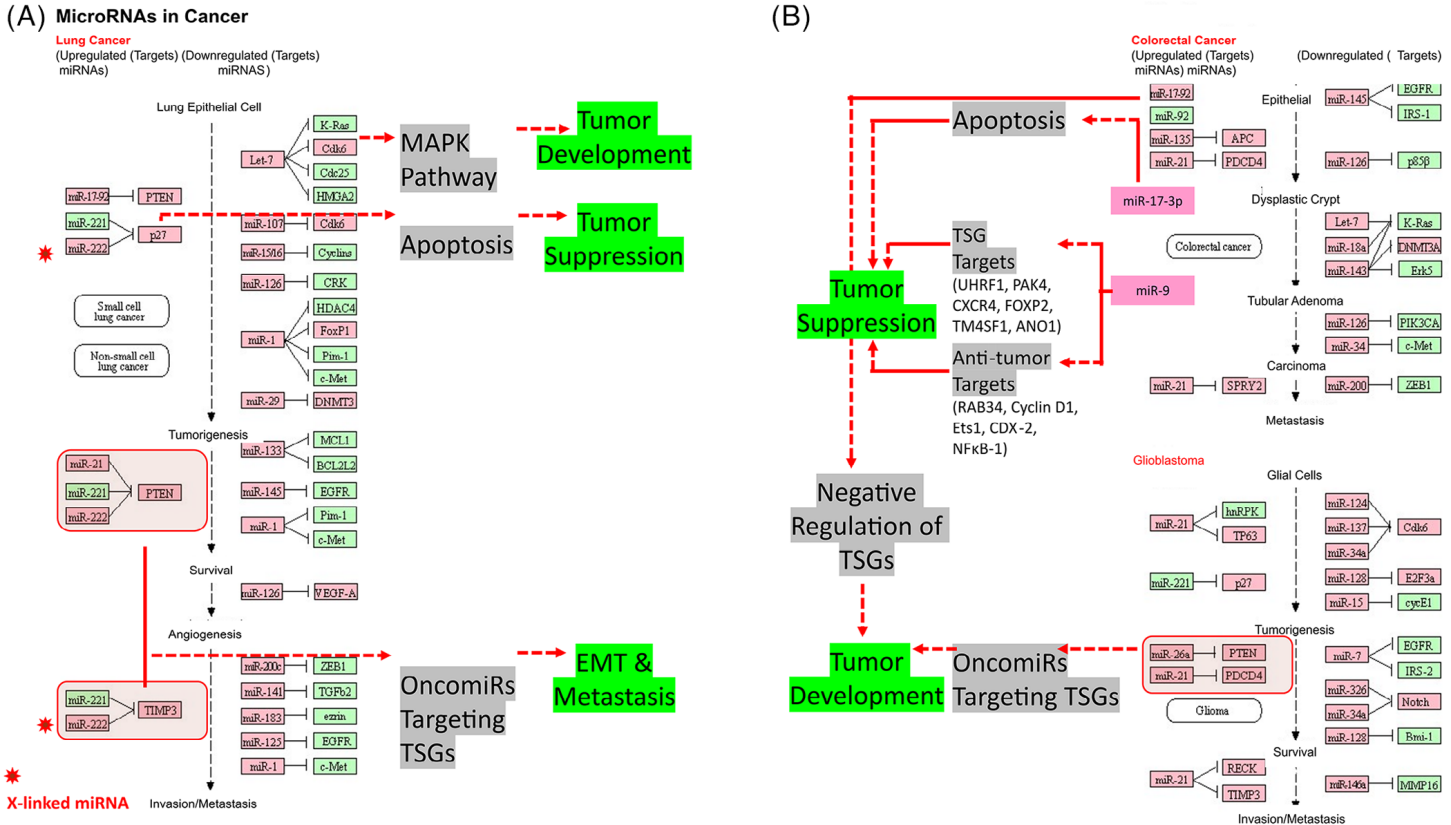
KEGG pathway—onco‐microRNAs in cancer (hsa05206) displaying TSGs in Homo sapiens in different cancers: (A) breast (left panel) and (B) colorectal cancer (right upper panel) and glioma (right lower panel) showing targeted pathways.

The investigation into the genetic basis of cancer has elucidated that a significant proportion of cancer cases can be ascribed to noncoding areas of the genome. Based on their respective lengths, ncRNAs can be categorized into two primary groups: short ncRNAs and lncRNAs. Small ncRNAs are typically characterized by their length, which is typically fewer than 200 nucleotides. This category includes several types of ncRNAs, including as small‐interfering RNAs (siRNAs), piwi‐related RNAs, transfer RNAs, and miRNAs. miRNAs attach to certain mRNA sequences located in the 3′UTR region and elicit either translational inhibition or mRNA destruction (Figure 6). Recent research works have provided evidence suggesting that miRNA may have a significant impact on human cancer. Specifically, miRNA has been found to target oncogenes (OCGs) or TSGs in order to regulate gene expression (Figure 7). When miRNA assumes an oncogenic function, it selectively targets TSGs, resulting in the initiation and progression of tumorigenesis (Figure 8). Conversely, if miRNA assumes the function of a tumor suppressor, it would selectively target OCG and inhibit the growth of tumors (Figure 8). Numerous distinct ncRNA sequences are present throughout cellular systems. Research conducted in the last 10 years has significantly changed our understanding of ncRNAs. Previously seen as insignificant byproducts of transcription, ncRNAs are now recognized as important regulatory molecules that have a role in several biochemical pathways such as chromatin and posttranscriptional alterations, and signal transmission. The involvement of ncRNAs can impact various molecular targets, leading to the regulation of distinct cellular responses and outcomes.

As we know, ncRNAs play a crucial role in governing physiological processes, as well as influencing mental and disease‐related conditions. ncRNAs have become evident as significant contributors in the field of cancer research, namely in their role as oncogenic drivers and their association with tumors across many types of cancer. The emerging targeted cancer treatments are based on small molecule inhibitors.^189^ Small molecule inhibitors decrease target protein function by attaching to their surface “pocket.” Small molecule inhibitors can bind more extracellular and intracellular targets than antibodies due to their size. Antibodies are subcutaneous or intravenous, but most small molecule inhibitors are oral. Additionally, some small molecule inhibitors can cross the blood–brain barrier to regulate intracranial lesions.^189^, ^190^, ^191^ This method of investigating tumor suppression offers possibilities for alternative treatments that could potentially restore the expression of TSGs to their normal levels, such as oligonucleotide therapy. Oligonucleotide treatments entail the introduction of external nucleic acids to regulate the activity of particular native genes. This study reviews two types of activating oligonucleotide therapy, namely small‐activating RNAs and synthetic mRNAs, as innovative approaches to enhance the expression of TSGs in cancer.

## 5 X‐LINKED TSG MUTANTS, VARIANTS, AND POLYMORPHISMS

Synonymous and nonsynonymous mutations contribute to the development of numerous human diseases and can be associated with the clinical outcome or responsiveness to therapy. Within the context of cancer, it is believed that these mutations account for a considerable percent of all driver mutations that develop as a result of single nucleotide substitutions. OCGs exhibit a higher prevalence of synonymous substitutions, but no indications of selection are observed in TSGs, except in *TP53*.^192^ Therefore, it is crucial to evaluate the current cutting‐edge computational techniques for predicting harmful synonymous mutations in order to enhance the present procedures and enhance performance. As a result of these gene alterations, several pathways are disrupted in cancer. According to Ref. 25, these genes are involved in a number of pathways that involve important lipid and protein kinases that function in cell growth and survival, the cell cycle process, DNA repair pathways, and cell death processes. Two types of cancerous mutations can be distinguished: (2) mutations in differentiation and apoptosis genes, such as AML/ETO and PML/RARa fusions, MLL rearrangements, mutations in *CEBPA*, *CBF*, *HOX* family members, CBP/P300, and coactivators of TIF1.^193^ Male *FOXP3* is a X‐linked TSG associated with the prostate. Because of the “single genetic hit” inactivation‐mediated carcinogenesis in men, somatic mutation or gene inactivation induce prostate cancer.^23^

### 5.1 Role of somatic mutations and nonsynonymous polymorphisms

Recent entire genome‐wide scan assessments have found a high number of somatic driver mutations in suspected cancer‐related genes and have supplied significant information regarding X‐linked cancer‐related genes.^194^, ^195^, ^196^ These driver mutations are favorably chosen during carcinogenesis and have been postulated to contribute to the neoplastic process. It is interesting to note that many X‐linked genes typically have driver mutations, suggesting that there may be other X‐linked TSGs. The X‐linked cancer‐related genes *FLNA*, *PFC*, *PRPS1*, *TARD8*, *MAGEE1*, *TAF*, and *KLH4* have all been associated with breast cancer.^195^, ^196^ X‐linked *FLNA*, *TBX22*, *KIAA2022*, *IRS4*, *PCDH11X*, *GPR112*, and *F8* have been proposed as cancer‐related genes in colorectal cancer. The X‐linked cancer‐related genes that have been associated with melanoma include *ZNF280C*, *IL3RA*, *PNMA3*, *NHS*, and *FGD1*. Clarifying the function of these genes in tissue‐specific carcinogenesis will help us better comprehend the process by which X‐linked TSGs are single‐hit inactivated during the onset and development of cancer. We include numerous characteristics, details of known & verified X‐linked TSGs (Table 1). A number of X‐linked cancer‐related genes, including *FLNA*, *PFC*, *PRPS1*, *TARD8*, *MAGEE1*, *TAF*, and *KLH4* have been associated with breast cancer.^194^, ^195^, ^196^ The X‐linked cancer‐related genes *FLNA*, *TBX22*, *KIAA2022*, *IRS4*, *PCDH11X*, and *GPR112* are connected to colorectal cancer. *SRPX*, *FOXP3*, *ZNF280C*, *IL3RA*, *FLNA*, *PNMA3*, *NHS*, and *FGD1* are X‐linked cancer‐related genes in melanoma.^22^

A number of studies conducted recently to decipher full genome‐wide scan assessments that revealed significant information about X‐linked cancer‐associated genes and discovered a large number of somatic mutations in putative cancer‐associated genes.^194^, ^195^, ^196^ These driver mutations, which are favorably chosen for during tumorigenesis, have been postulated to contribute to the neoplastic process. It is interesting to note that many X‐linked genes typically contain somatic mutations, which could indicate the presence of additional X‐linked TSGs.^197^ One of the most prevalent types of genetic variations found in the human genome is single nucleotide polymorphisms (SNPs). Genetic vulnerability to carcinoma is linked to SNPs in the genes that control DNA repair, cell cycle regulation, metabolism, and immunity.^198^, ^199^, ^200^ Understanding the molecular pathologies in various cancers requires a depth insight of the mechanistic basis of the effects of polymorphisms that increase cancer vulnerability. SNPs may be potential diagnostic and therapeutic biomarkers in several cancer types from a clinical standpoint. Genes contain SNPs in a variety of locations, including the promoter, exons, and introns as well as the 5′‐ and 3′ UTRs. SNP location affects gene expression and its effect on cancer susceptibility. When SNPs in the promoter region modify promoter activity, transcription‐factor binding, DNA methylation, and histone modifications,^201^, ^202^, ^203^, ^204^, ^205^ the resulting changes in gene expression are known as “promoter‐region effects.” If exonal SNPs reduce cistron transcription and translation, they may increase the risk of tumorigenesis.^206^, ^207^, ^208^ If SNPs situated on intron regions produce alternative transcript splice forms and either enhance or impair lncRNA binding and function.^209^, ^210^, ^211^ The translation is impacted by SNPs in the 5′‐UTR, but miRNA binding is impacted by SNPs in the 3′‐UTR.^212^, ^213^, ^214^ If SNPs in distant regions from specific genes have long‐range cis effects that either decrease or increase gene transcription.^200^, ^215^ Through cis‐acting elements and trans‐acting factors, the promoter region controls the onset and pace of gene transcription. TF binding is impacted by promoter‐related polymorphisms, which changes promoter activity, gene transcription, mRNA stability, and translation. After that, these effects change protein levels, which likely confirms the person's propensity for diseases like cancer. Additionally, variations in the promoter regions impact epigenetic processes including DNA methylation and histone alterations, which in turn affect cancer risk. The amino alkanoic acid substitution caused by nsSNPs can change how proteins function. In most cases, modifications to a codon's first two bases result in modifications to amino alkanoic acids. By altering number of H‐bonds and posttranslation modifications, changes in the amino acid sequence will change the secondary structure of the protein, which will change how the protein interacts and performs. As a result, these modifications affect levels of protumor and tumor suppressor proteins in addition to cell signaling pathways. Because of modifications to the structure and functionality of the encoded proteins, nsSNPs have an effect on cancer susceptibility.

### 5.2 Role of synonymous polymorphisms in cancer development and progression

Synonymous SNPs (sSNPs) have long been regarded as functionally inert, although there have been a few instances of cancer‐causing sSNPs documented.^216^, ^217^ Precise detection of driver mutations is essential in genomic investigations of human malignancies. Although many missense mutations that induce cancer have been discovered, the investigation of putative cancer drivers for synonymous mutations has yielded modest progress thus far. Recently, Cheng et al.^216^ developed a new and innovative machine learning framework called epSMic. Interestingly, its purpose is to accurately forecast synonymous mutations (that does not entail change in protein sequence) that drive cancer. The epSMic utilizes an iterative feature representation approach that enables the acquisition of discriminative features from different sequential models in a supervised iterative manner. Cheng et al.^216^ created the benchmark datasets and encoded the embedding sequence, physicochemical property, and basic information including conservation and splicing characteristic. The evaluation results on benchmark test datasets indicate that epSMic surpasses current approaches, rendering it a viable resource for researchers in detecting functional synonymous mutations in cancer.

To enhance our ability to make accurate predictions, Cheng et al.^218^ created an ensemble model called Prediction of Deleterious Synonymous Mutation (PrDSM). This model combines the scores produced by the three most precise predictors. The results of their benchmark testing showed that the ensemble model PrDSM performed better than the reviewed tools in predicting detrimental synonymous mutations. Similarly, Zeng and Bromberg^217^ obtained four kinds of single nucleotide variants (sSNPs) from the databases: germline variants, somatic variants in normal tissues, somatic variants in malignant tissues, and probable cancer drivers. Their research discovered that by examining single nucleotide variations (sSNPs) for their frequency among patients, evaluating the conservation of the specific genomic location they affect, and using synVep prediction (a machine learning‐based tool for predicting the effects of sSNVs), they were able to identify cancer driver variants (referred to as proposed drivers) and previously unidentified potential cancer genes. Out of the 2.9 million somatic single nucleotide variants (sSNPs) recorded in the COSMIC database, they identified 2111 sSNPs that are suggested to be cancer driver mutations. Out of these, a total of 326 single nucleotide variants (sSNPs) were identified that could potentially have an impact on RNA splicing, RNA structure, and RNA binding protein motifs. This compilation of hypothesized cancer driver sSNPs offers computational assistance in prioritizing the experimental assessment of synonymous mutations detected in cancer cases. In addition, the compilation of novel potential cancer genes, stimulated by synonymous mutations, could bring attention to previously un‐investigated cancer pathways.

## 6 X‐LINKED TSGS MEDIATE IMMUNE RESPONSE AND CONTRIBUTE TO THE GENDER‐BASED DISCREPANCIES IN DIFFERENT CANCERS

X chromosome has the most immune‐related genes in the human genome. For this reason, the X chromosome has garnered attention and many research have sought to understand how genes on it cause and perpetuate diseases.^219^

### 6.1 Immunomodulatory role of TSGs

The tumor immune microenvironment (TIME) and tumor cells exhibit a mutually influential and interconnected connection. As the tumor advances, the tumor cells form an immunosuppressive environment, enabling them to evade immune detection. Concurrently, an immunosuppressive factor called TIME facilitates tumor growth by depleting T cells that infiltrate the tumor, activating immune checkpoint genes including VISTA, TIM‐3, and LAG‐3 that hinder immune response, and inhibiting immune cells such as Tregs, TAMs, and myeloid‐derived suppressor cells (MDSCs).^220^ To enhance the efficacy of cancer treatment methods, it is imperative to investigate both tumor cells and the immunological microenvironment of the tumor. There exist an obvious positive and negative association between OCGs, TSGs, immune cells, and their infiltration in the TME. Zhao et al.^221^ conducted a study to identify genes that are expressed differently and are implicated in the impact of antiangiogenic therapy on the infiltration of MDSCs, and to examine the mechanisms by which these genes function. The cytoHubba plugin of the Cytoscape platform was used to perform PPI network analysis and identify the top 10 important genes. The cytoHubba plugin of Cytoscape identified the following 10 important genes with the highest degree scores: AURKB, RRM2, BUB1, NUSAP1, PRC1, TOP2A, NCAPH, CENPA, KIF2C, and CCNA2. The majority of these genes exhibited increased expression in LUAD and were linked to the infiltration of immune cells and the prognosis of malignancies. An examination of the connections between differentially expressed genes (DEGs) and the infiltration of specific immune cells has identified consistent patterns in the genes that are downregulated. As per their results, these genes show positive associations with the levels of Th2 cells, γδ T cells, and CD56dim NK cells, while displaying negative associations with other infiltrating immune cells. Wang et al.^222^ developed the gene signature based on lactate, that was highly successful in predicting the prognosis and regulating the tumor microenvironment (TME) in diffuse large B‐cell lymphoma (DLBCL). Their findings highlights the significance of the lactate gene, STAT4, as a crucial tumor suppressor in DLBCL. This suggested that targeting STAT4 modulation shows potential as a viable approach for treating DLBCL in a clinical setting.

### 6.2 X‐linked TSGs, gender disparity, and cancers

X chromosome may considerably sex bias in illness susceptibility.^3^ XCI is regulated by the XIC and contains immune‐related genes and regulatory elements.^223^ Therefore, the X chromosome is important in the immunological response, and genes that escape inactivation or are preferentially inactivated may affect the dosage of X‐linked gene expression between sexes and so disease sex bias. It is crucial to study XCI mechanisms to comprehend all regulatory aspects including sex bias. Considerable emphasis is placed on genes that are located on the X chromosome, as opposed to autosomes, as they contribute to the gender‐specific discrepancy observed in various cancer types. The prevalence of different cancer forms in males and females can be largely attributed to sex‐specific variances, as discussed in Ref. 224. According to Bray and coauthors, lung cancer is the prime reason of deaths among males, with prostate, colorectal, liver, and stomach cancer following closely behind. Conversely, breast cancer exhibits a higher incidence rate among females. According to a study conducted by Siegel et al.,^225^ it was shown that the rates of cancer incidence were 20% greater in males compared with females.

Sexual dimorphisms in cancer development have been attributed to evolution, heritable traits, sex hormones and chromosomes, and environmental carcinogens.^226^ While all models contribute to carcinogenesis, the role of sex chromosomes and hormones (estrogens and androgens) in male versus female immune cell responses in tumor growth has not been fully investigated. Inadequate systems and experimental instruments have also caused confusion. This review aims to clarify the functions of sex bias in cancer adaptive immunity. Estrogens (greater in females) enhance survival cytokines, promote immunoglobulin excretion, and modify T cell activity, while androgens (higher in males) inhibit antibody formation, increase anti‐inflammatory cytokines, and limit T cell proliferation.^227^, ^228^ To identify its significance in the innate and adaptive immune response and how it differs between sexes, the X chromosome should be carefully studied. We believe that studying the X chromosome's function and integrating it in biological analysis could help us understand complex pathologies such as cancers.

## 7 IMPLICATIONS IN CANCER, PROGNOSIS, AND CLINICAL RELEVANCE

The viability of numerous healthy cells relies on signals from growth factors or the extracellular matrix, which inhibit apoptosis. On the other hand, tumor cells can often survive without the growth factors that are necessary for the survival of normal cells. The inability of tumor cells to undergo apoptosis in the absence of normal environmental cues is significant not only in the initial formation of tumors, but also in the survival and expansion of metastatic cells in aberrant tissue locations. Normal cells undergo apoptosis, a process of programmed cell death, in response to DNA damage. However, many cancer cells do not undergo apoptosis in the same way. In this scenario, the lack of apoptosis has a role in the resistance of cancer cells to radiation and certain chemotherapy medications, which function by causing damage to DNA. Aberrant cell viability, together with cell replication, thus, significantly contributes to the relentless expansion of cancer cells in an organism.

### 7.1 Implications in cancer initiation, progression, and metastasis

It is well acknowledged that cancer is a genetic disorder, with two primary types of genes, namely OCGs and TSGs, playing a crucial role in the development of cancer. The MYC OCG undergoes chromosomal translocation, leading to the formation of N‐MYC, C‐MYC, and L‐MYC proteins. These proteins are encoded by proto‐OCGs located on chromosomes 2, 8, and 1. The MYC gene regulates the cell cycle through a nuclear DNA‐binding protein, promoting transformation, dedifferentiation, immortalization, and cell proliferation.^229^ The primary cause of human pancreatic cancer is typically a single missense mutation in the KRAS gene, specifically affecting the 12th codon and resulting in a substitution of valine or aspartate for glycine, ultimately leading to protein activation. Pancreatic tumor development and onset entail the activation of oncogenic KRAS through metastasis, metabolic changes, treatment resistance, and modifications in signal transduction pathways. In distal metastasis, the rapid regression of the tumor might occur when advanced or precursor lesions of KRAS vanish.^230^, ^231^, ^232^ Tumor start, maintenance, and progression are all influenced by the presence of oncogenic KRAS.^233^

NRAS, HRAS, and KRAS are closely correlated. KRAS encodes a small membrane GTPase involved in KRAS signaling. RAS is activated by interacting with GTP in the presence of guanine exchange factors (GEFs). RAS is transformed to its inactive state through hydrolysis of GTP to GDP, facilitated by GAPs. RAS proteins stimulate several biological activities, such as cell proliferation, through multiple downstream signaling pathways. Each member of the RAS family has distinct activity on various cell types through gene regulation. KRAS is essential for the progression of pancreatic, colon, and lung cancer due to its ability to induce GTP to GDP hydrolysis following oncogenic mutations that activate RAS. Various types of tumors exhibit distinct control of KRAS.^234^

Stimulation of a receptor‐linked tyrosine kinase, like the epidermal growth factor receptor, can trigger the activation of RAS in a signaling cascade located upstream. Phosphorylation cascade can be triggered by the homodimerization of epidermal growth factor receptors when they bind to external growth factors. Subsequently, GEFs and growth factor receptor‐bound 2 adaptor protein activate RAS by facilitating the exchange of GDP for GTP, resulting in the creation of KRAS‐GTP. Rapidly accelerated fibrosarcoma (RAF) serine/threonine kinases are drawn to the cellular membrane and activated by KRAS‐GTP. RAS homolog enriched in brain (RHEB) is inhibited by a protein complex formed by many genes, including TSC1 and TSC2, following activation by ERK. The activation of the PI3K enzyme, composed of the p110 subunit (consisting of p110 and p85), is triggered by KRAS‐GTP. This activation initiates the PI3K signaling pathway, resulting in the conversion of phosphatidylinositol‐4,5‐bi‐phosphate into phosphatidylinositol‐4,5‐tri‐phosphate (PIP3). AKT can activate either mechanistic target of rapamycin kinase complex 2 (mTORC2) or phosphoinositide‐dependent kinase‐1 (PDK1) on the plasma membrane following its stimulation by PIP3. PTEN, a negative regulator of adenylate kinase 3, is suppressed by the activation of nuclear factor kappa‐light‐chain‐enhancer of activated B‐cells (NF‐kB) through the action of AKT. Furthermore, mTORC1 contains the inhibitory TSC1/TSC2 complex, which is deactivated by AKT. Binding of GTP to the tiny RAL A/B GTPases activates them through the KRAS‐GTP activation of RAL‐GEF. TANK inhibits NF‐kB release by binding to TANK‐binding kinase 1 when RAS‐related protein B (RALA/B) is present. Protein translocation, cell proliferation, and cell differentiation are influenced by the three signaling cascade branches.^233^

Several medications have been developed to specifically target hyperactive OCGs, including kinase inhibitors like sorafenib for HCC and imatinib for chronic myeloid leukemia.^235^, ^236^ Nevertheless, there has been a noticeable deficiency in small molecule medications that specifically target TSGs that are either underactive or altered. The active small membrane GTPase RAS is crucial in the KRAS pancreatic signaling pathway, which triggers the activation of the PI3K, RAF/MEK/ERK, and RALA/B signaling pathways. Numerous direct and indirect connections exist among these branches, along with multiple inhibitory and regulatory feedback loops. Instances exist when this phenomenon has occurred, such as the detection of thioemicarbazone family compounds in their ability to selectively bind to mutant p53, facilitating the removal of zinc ions and subsequently restoring the DNA‐binding capabilities of this crucial transcription regulator.^237^ In addition, chemicals like PhiKan083 facilitate the reinstatement of regular functionality in p53 mutant cells that possess a Y220C or Y220S mutation by enhancing the stability of the protein structure and inhibiting its denaturation.^238^, ^239^ Nevertheless, despite substantial study into the structure and function of the p53 gene and protein, no medications specifically designed to target p53 have successfully completed clinical trials and gained approval. This highlights the challenges associated with targeting TSGs. APR‐246, a quinuclidinone derivative, is a p53 activator now undergoing clinical trials. It functions by binding with the cysteine residues within the protein, thereby restoring the wild‐type conformation and rescuing the capacity of mutant p53 to connect with DNA.^240^ APR‐246 has successfully concluded phase I and II clinical studies for various cancer types, both as a standalone treatment and in conjunction with other anticancer medicines.^241^, ^242^, ^243^ The initial human trial demonstrated that the medication exhibits encouraging antitumor effects, as evidenced by heightened apoptosis of circulating malignant cells in certain individuals, irrespective of TP53 mutant status. Nevertheless, the medicine APR‐246 undergoes rapid degradation under normal bodily settings, which hampers its efficacy as a standalone treatment. This degradation occurs due to the conversion of APR‐246 into its active form, methylene quinuclidinone.^244^

### 7.2 Diagnostic and prognostic applications

The TSGs may offer novel molecular signals linked to the formation of tumors and the progression of cancer and could have significant clinical consequences for the detection, prediction, and management of cancer.^3^ The TSGs mostly participated in pathways connected with metabolism, immunological response, and cell growth signaling. Several TSGs exhibited a good link with survival prognosis in different types of malignancies, thereby validating their role in suppressing tumor growth. Sun and coauthors identified 17 TSGs that exhibit considerably elevated expression levels in the small intestine compared with other gastrointestinal (GI) tissues.^222^ Several master transcriptional regulators (MTRs), including HNF4A, ZBTB7A, p53, and RUNX3, were identified as tumor suppressors. Additionally, these genes demonstrate dramatically reduced expression levels in GI malignancies compared with normal tissues. They showed that the decrease in expression of several TSGs is linked to their increased methylation in cancer. Furthermore, they demonstrated that the levels of expression of numerous TSGs were inversely associated with tumor purity and positively associated with the immune response against tumors in different types of cancer. This suggests that these TSGs may act by enhancing the body's immune response against tumors, hence suppressing their growth. In addition, they discovered a transcriptional regulatory network consisting of the TSGs and their MTRs. EIF4A3 is a newly discovered protein that acts as a suppressor of m6A, a type of mRNA modification. It has the ability to regulate the overall level of m6A modification in mRNA, which in turn affects the fate of genes. Although there is growing evidence indicating the significant involvement of EIF4A3 in both tumor progression and immunity, a thorough analysis of EIF4A3 across different types of cancer has not been carried out. This analysis is necessary to determine whether EIF4A3 can serve as a reliable biomarker for cancer screening, prognosis prediction, and to aid in the development of precise therapies for different types of cancer.^245^Sperm‐associated antigen 6 (SPAG6) has been recognized as either an OCG or a tumor suppressor in different forms of human cancer. Nevertheless, the specific function of SPAG6 in BCR::ABL1 negative myeloproliferative neoplasms (MPNs) has been clearly elucidated by Ding and coauthors.^245^ In the study, they discovered that the expression of SPAG6 was increased at the mRNA level in primary cells from patients with MPNs and in cell lines derived from MPN‐associated malignancies. Hence, SPAG6 could be used as a diagnostic marker in MPNs. The APRO (antiproliferative protein) family, which consists of six members (TOB1, TOB2, BTG1, BTG2, BTG3, and BTG4), encodes trans‐membrane glycoproteins that have been recently discovered. It has been claimed that the APRO family is linked to the onset and advancement of cancer. The objective of this study is to conduct a thorough examination of the APRO family of proteins as a predictive biomarker in different types of human malignancies. Zhang and coauthors conducted a comprehensive investigation of the APRO family across multiple types of cancer using data from TCGA. Using bioinformatics techniques, they investigated the predictive significance of the APRO family and the relationship between APRO family expression and tumor mutation burden, microsatellite instability, drug sensitivity, and immunotherapy in several types of cancer. Their findings indicate that the APRO family was predominantly suppressed in the cancer samples. The expression of APRO family members was associated with patient prognosis. Furthermore, the APRO family genes exhibited a noteworthy correlation with immune infiltration subtypes, TME, and tumor cell stemness.^246^ Their investigation conclusively established the correlation between APRO family genes and medication sensitivity. Their study also offered extensive data to comprehend the significance of the APRO family as an OCG and prognostic indicator in certain types of tumors.^246^

### 7.3 Clinical relevance

Antioncogenic therapy is being researched in breast cancer using antisense sequences targeting c‐fos and c‐myc. Many other genes associated with breast cancer are being focused on utilizing antisense methods in preclinical studies.^247^ Research has explored the use of gene therapy including the adenoviral E1A gene, which interacts with the HER‐2 promoter to reduce its expression, as a potential treatment for cancer. A phase I clinical trial including intracavitary liposome‐complexed E1A gene in breast and ovarian cancer showed decreased HER‐2 protein levels, enhanced E1A expression in cancer and normal cells, reduced pleural or peritoneal cancer cells, and increased apoptosis.^248^ A phase II experiment is now ongoing.

An alternative strategy employed to overcome the reduced activity of TSGs is focusing on the subsequent effects resulting from their decreased expression. For instance, when *PTEN* is downregulated or lost, it results in excessive activity of the AKT pathway. This makes the kinase AKT an excellent target for small molecule medicines by inhibiting its function.^249^ Nevertheless, oligonucleotide treatments provide a distinct benefit by directly targeting the underlying cause, specifically the underactive TSGs. Given the significant impact of TSG regulation on different types of cancer, there is much enthusiasm for the possibility of selectively targeting certain TSGs as a new therapeutic strategy for patients. This review examines the prospect of utilizing oligonucleotide therapeutics to reinstate the function of TSGs in cancer, as well as the advantages and difficulties associated with this strategy.

## 8 DISCUSSION

According to earlier studies,^250^, ^251^, ^252^ X‐linked genes may play a role in cancer. However, distinct some malignancies result from specific gene present on the X chromosome.^106^ According to Ref. 197, the most of X‐linked TSGs are connected to the tumorigenesis of breast cancer. According to Ref. 147, breast cancer is caused by genomic and epigenomic alterations in healthy host cells. In this article, we present a database summary of the X‐linked TSG mutation patterns and epigenetic alterations. According to some studies, X‐linked TSGs survive X chromosome deactivation and may play a role in cancer.^253^, ^254^ In hybrid cell lines that still include a human Xi, the human genes exhibit 15% X‐linked genes that escape XCI. Male and female cancer are greatly affected by TSG mutations that avoid XCI.^253^, ^255^ The human X chromosome's short arm is where the escaping genes are typically found in clustered form.^116^, ^256^

Current whole genome‐wide scan analyses have found an increased frequency of somatic mutations in different cancers^194^, ^195^, ^196^ and provide important information about X‐linked cancer. It is fascinating that 19 X‐linked TSGs that are connected to breast cancer have been discovered. *DUSP9*, *FLNA*, *GPC3*, *RBBP7*, *KDM6A*, *RBMX*, *FOXP3*, and *EDA2R* mutation patterns are among them and are commonly discovered.^257^, ^258^, ^259^ Researchers have found that epigenetic alterations, in addition to gene mutation, are critical in the development of tumors. Numerous studies have linked breast cancer to the epigenetic silencing of autosomal genes like *RASSF1* at locus 3p21.31^260^; *NDRG1* at locus 8q24.22.^261^, ^262^ Globally, it has been determined that cancer cells have hypomethylated genomic DNA, which directly stimulates OCGs,^263^ and hypermethylated DNA, which promotes the spread of cancer by inactivating TSGs.^106^ DNA methylation and GC content displayed a favorable correlation.^264^ In comparison with GC‐poor regions, the GC‐rich regions offer more potential targets for methylation and exhibit an elevated frequency of methylated sites.^265^ In the investigation, we identified that genes’ ±1 kb areas had roughly comparable percentages of GC content. A study examined 23 X‐linked TSGs and discovered that there were direct and indirect relationships between them based on the GeneMANIA network.^3^ Coding X‐linked TSGs communicate with autosomes and X‐linked TSGs. Multiple pathways are dysregulated in breast cancer as a result of X‐linked TSG mutation and epigenetic alteration. These include the cell cycle machinery, DNA damage response pathways, apoptosis, and pathways involving important lipid and protein kinases involved in cell growth and survival.^266^, ^267^ Breast cancer is brought on by the downregulation of the ERK and activation of the p38 signaling pathways through *DUSP9*,^268^ inhibition of the MAP/ERK signaling pathway through *FLNA*.^269^ To the best of our knowledge, no one has discussed the methylation‐related gene silencing in X‐linked TSGs that is related to breast cancer. However, hypo‐hypermethylation of X‐linked TSGs results in tumorigenesis and genomic instability, which can result in a variety of cancers, primarily breast cancer. We uncovered the functional relationship between autosomes, X‐linked TSGs, and noncoding X‐linked TSGs such as miRNAs.

## 9 CONCLUSION

On many aspects, cancer is complicated. The molecular phenomenology of cancer is also rich. Carcinogenesis involves a complex process of genetic changes that impact crucial cellular pathways related to growth and development. OCGs are genes that, when altered, result in increased activity, whereas TSGs, when altered, lead to decreased activity, both of which contribute to the development of cancer. The impacts of these modifications are intricate because of the numerous changes in a standard case of breast cancer and the interconnections of the biological pathways involved. The mutational and genomic origins of cancer and their downstream impacts on mechanisms like gene regulatory control reprogramming and molecular pathways dependent on such regulation are key to defining the etiology and pathophysiology of the disease. More crucial is to know their origins, prognosis, and treatments. Cancer gene expression abnormalities are caused by several reasons. Bert Vogelstein's group concluded, based on high‐throughput sequencing of numerous human cancer cell genomes, that a limited number of proto‐OCGs (*n* = 55) and TSGs (*n* = 70) are responsible for the majority of recurrently mutated drivers of OCGsis in the most prevalent and deadly forms of human cancer. Nevertheless, there are still unidentified genes that could potentially function as drivers in uncommon forms of cancer and potentially as infrequent drivers in more prevalent forms of cancer. Experiments using various omics technologies may now study several of these aspects. However, characterizing each dimension separately has failed to provide a complete picture of expression control. In this review, we examine some of the most important aspects pertaining to X chromosome–autosome crosstalk with implications in different cancers. We have made a thorough discussion from X‐linked genes discovery to mechanisms regulating their gene expression in normal and tumor situations. We also presented the how X chromosome–autosome crosstalk are influenced by changes in the epigenome and mutational landscape of the disease with an emphasis on ncRNAs (especially miRNAs).

The cancer metastatic cascade is an intricate process involving various elements that contribute to the dissemination and proliferation of cancer cells at secondary sites. During this intricate process, multiple genes have been recognized as metastasis suppressors, functioning to impede the occurrence of metastasis. Notably, certain genes have demonstrated involvement in the regulation of the TME. This review discusses the latest advancements in the field of metastasis suppressor genes and their interaction with the microenvironment.

Unfortunately, there are not many noncoding gene/region databases, which makes it challenging to comprehend how ncRNAs may affect cancer. While it has been shown in numerous studies that miRNAs can bind to various target sites, the regulation mechanism of mRNA‐lncRNAs in TSGs is still unknown, and some of its proposed applications are not yet widely acknowledged. Our review will help the scientific community better understand the X‐linked TSGs' genomic preference, methylation‐related epigenetic changes, functional genomic conservation, ncRNAs, and TFs nearby, as well as nonsynonymous SNPs in a few X‐linked TSGs. This will help us better understand how X‐linked TSGs and sex chromosomes, or autosomes, interact under the influence of diverse range of miRNA regulatory networking. An intricate web of gene regulatory mechanisms regulates how genes are expressed. Several ncRNAs control gene expression either before or after transcription, depending on the sequence. X‐linked TSGs mRNAs are targets for miRNAs because they stimulate mRNA degradation and repress gene translation or transcription, which leads to the breakdown of TSGs that support different forms of cancer.

To understand cancer's complex gene expression dysregulation in relation to X‐linked TSGs, we contextualize everything in a systems biology‐based multiomics regulatory framework. Well‐researched OCGs and TSGs may not always be the most optimal targets. The list of OCGs and TSGs has grown due to the increasing amount of genetic and genomic data in cancer, as a result of various studies involving molecular, cellular, genomic, and computational analysis, which also include ncRNA genes.^270^, ^271^ OCG mutations enhance function while TSG mutations reduce function, suggesting that TSGs and OCGs may regulate cellular processes in an opposing yin‐yang manner.^272^ Discovering genes poses a significant barrier to high‐throughput, large‐scale methods, mostly because of the statistical and bioinformatics limitations in effectively evaluating vast amounts of data. Examples are already emerging. Through comparative genomic hybridization, detailed information on gene copy numbers on chromosome 22q13 showed that a big amplicon was really composed of several regions with gene amplification and gene deletion.^273^ Functional testing confirmed that the amplified *ZNF217* gene encodes a TF capable of immortalizing human mammary cells,^274^ while CYP24 encodes a protein that hinders the prodifferentiating effects of vitamin D,^275^ both of which are characteristics of OCGs. The putative OCGs can be confirmed using gene knockout experiments and targeted with small molecules, antibodies, or gene therapy.

Current gene therapy trials involve the transfection of immune modulators like antigens and cytokines, as well as the protection of stem cells using the multidrug resistance gene. Stem cells are also purged using the proapoptotic bcl‐xs gene or the herpes virus thymidine kinase gene along with gancyclovir. Efforts have also been made to develop techniques for replacing the BRCA‐1 gene. The intraperitoneal delivery of the retroviral LXSN‐BRCA1 vector in BRCA‐1‐deficient ovarian cancer did not result in any clinical responses, possibly because of the vector's instability in the body.^276^ Currently, there are no ongoing applications of BRCA‐1 or BRCA‐2 gene replacement in breast cancer.

A novel genome engineering technology called clustered regularly interspaced short palindromic repeat (CRISPR)‐associated protein‐9 (Cas9), which contains an RNA domain‐containing endonuclease, has been demonstrated as an effective method for treating cancer cells. This technique is powerful due to its multifunctional properties such as high specificity, accuracy, time efficiency, cost‐effectiveness, and minimal off‐target effects. Hazafa et al.^277^ reviewed recent studies on the recently developed genome‐editing technique, CRISPR/Cas9, as a promising preclinical therapeutic approach for reducing and identifying novel target genes in solid tumors. Their analyses and data showed that CRISPR/Cas9 effectively slowed down the growth of various tumor cells (breast, lung, liver, colorectal, and prostate) by targeting specific genes related to cancer development and treatment. This suggests that CRISPR/Cas9 has the potential to be a valuable therapeutic tool in inhibiting tumor growth by reducing cell proliferation, metastasis, invasion, and promoting cell death in cancer treatment. The latest findings indicate that the CRISPR/Cas9 tool efficiently targets DNA, leading to the temporary or permanent deactivation of genes. This makes it the superior genome editing technology with less off‐target effects compared with the RNAi technique.^278^, ^279^

This review discusses prevalent anomalies in OCGs and TSGs in different human cancers and their established correlations with tumor categorization, prognosis, and response to particular treatments. Enhanced comprehension of these connections has resulted in novel therapeutic uses. Therapeutic agents that focus on OCGs and their related pathways are currently being used in clinical settings, with numerous others in various stages of preclinical and clinical trials. The variety of antibodies, small synthetic chemicals, cytokines, gene therapy approaches, and natural compounds with specialized biological features has significantly expanded the pool of potential medications. However, therapeutic achievements have been restricted due to the overlapping of numerous cancer‐related pathways and the significant diversity in genotype and phenotype across individual tumors. Similarly, efforts to substitute TSG functions encounter many technical obstacles. This review provides an overview of the present advancements and future opportunities in targeting OCGs and TSGs for therapeutic purposes, as well as new technologies for improved tumor classification and prediction of responses to standard and innovative treatments. Future research should focus on overcoming problems related to the administration and immune response of oligonucleotide treatments. This will enhance their capacity to advance through clinical trials and effectively increase TSG expression in cancer. Moreover, with the advent of targeted oligonucleotide therapies in medical practice, the utilization of tumor genome sequencing could facilitate the identification of an optimal treatment for a patient, taking into account the genetic characteristics of their tumor. The scientific field is still in its early stages and offers promising prospects for advancement and practical use in the future. We envisage that, the understanding from this review will lead to new theories for the detection and management of cancer as well as insights into the functional relationships between OCGs, TSGs, miRNA, and other biomolecules toward precision medicine and personalized therapies.

## AUTHOR CONTRIBUTIONS

Tikam Chand Dakal and Amit Sharma conceived the idea and designed the structure of the review article. Tikam Chand Dakal, Bhanupriya Dhabhai, Anuja Pant, Kareena Moar, Kanika Chaudhary, Vikas Yadav, Vipin Ranga, Narendra Kumar Sharma, Abhishek Kumar, Pawan Kumar Maurya, Ingo G. H. Schmidt‐Wolf, and Amit Sharma wrote the review. Tikam Chand Dakal, Jarek Maciaczyk, Ingo G. H. Schmidt‐Wolf, and Amit Sharma revised the review article. All authors read and approved the manuscript and its submission.

## CONFLICT OF INTEREST STATEMENT

Authors declare that they have no conflict of interest.

## ETHICS STATEMENT

Not applicable.

## Data Availability

Not applicable.
